# Plant hormones and membrane transporters: integrating nutrient uptake, ion homeostasis, and stress responses through hormonal cross-talk

**DOI:** 10.3389/fpls.2025.1699212

**Published:** 2026-02-02

**Authors:** Mohammad Faizan, Jingdong Chen, Fadime Karabulut, Renuka Sharma, Shadma Afzal, Pooja Sharma, Pravej Alam, Shamsul Hayat, Ira Khan, S. Maqbool Ahmed, Xigang Dai, Heping Wan, Changli Zeng, Haider Sultan

**Affiliations:** 1 Hubei Engineering Research Center for Protection and Utilization of Special Biological Resources in the Hanjiang River Basin, College of Life Sciences, Jianghan University, Wuhan, Hubei, China; 2 Botany Section, School of Sciences, Maulana Azad National Urdu University, Hyderabad, India; 3 Bitlis Eren University, Hizan Vocational School, Bitlis, Türkiye; 4 Shree Guru Gobind Singh Tricentenary University, Gurugram, Haryana, India; 5 Department of Bioclimatology, Faculty of Environmental Engineering and Mechanical Engineering, Poznan University of Life Sciences, Poznań, Poland; 6 Department of Biotechnology, Baba Farid College of Engineering and Technology, Bathinda, Punjab, India; 7 Department of Biology, College of Science and Humanities, Prince Sattam Bin Abdulaziz University, Alkharj, Saudi Arabia; 8 Department of Pharmacognosy, Faculty of Pharmacy, Tishk International University, Erbil, Kurdistan Region, Iraq; 9 Plant Physiology Section, Department of Botany, Faculty of Life Sciences, Aligarh Muslim University, Aligarh, India

**Keywords:** hormonal regulations, membrane transporters, ion homeostasis, stress tolerance, phytohormonal balance

## Abstract

Plant membrane transporters are essential components in the regulation of nutrient uptake, ion homeostasis, and adaptive responses to environmental stress. These transport processes are tightly coordinated by complex phytohormonal signaling networks. This review provides an in-depth examination of the molecular mechanisms through which major plant hormones including auxins, cytokinins, brassinosteroids, ethylene, abscisic acid, gibberellins, jasmonates, strigolactones, melatonin, karrikins, and gamma-aminobutyric acid (GABA) modulate transporter activity. Each hormone activates distinct signaling pathways that alter the transcription, localization, and functional dynamics of membrane transport proteins, enabling plants to fine-tune physiological responses in accordance with developmental needs and environmental stimuli. Special attention is given to the integration of hormonal signals and how this interplay governs key processes such as stomatal movement, nutrient transport, and hormonal cross-regulation. The review also highlights the role of hormone–transporter crosstalk in optimizing plant performance under both normal growth conditions and various abiotic or biotic stresses. By dissecting these regulatory mechanisms, we offer insights into how phytohormonal control of membrane transport contributes to overall plant fitness. Understanding the coordination between hormonal signaling and transporter networks opens new avenues for crop improvement strategies. Leveraging this knowledge can support the development of resilient plant varieties with enhanced nutrient use efficiency, stress tolerance, and yield potential. This review underscores the significance of transporter–hormone interactions as central elements in plant development and environmental adaptation, positioning them as key targets for future agricultural innovations.

## Highlights

- Membrane transporters control nutrient uptake, ion homeostasis, and overall cellular functions.
- Phytohormones regulate transporters by modulating their expression, localization and activity.
- Interaction between hormone signaling pathways and transporter networks allows plant to adapt to both biotic and abiotic stresses.
- Integrating hormone-transporter interactions offers new strategies to enhance plant resilience and productivity.

## 1 Introduction

Membrane transporters are integral components of cellular membranes that mediate the selective translocation of ions, metabolites, phytohormones, and xenobiotics across cellular and subcellular boundaries, thereby maintaining physiological homeostasis and supporting plant growth, development, and adaptation to environmental stresses (Akhiyarova et al., 2024; Jiang et al., 2024; Yuan et al., 2024). These proteins are classified into several major families, such as ATP-binding cassette (ABC) transporters, the major facilitator superfamily (MFS), and various ion channels, each fulfilling distinct roles in nutrient acquisition, ion balance, detoxification, and intracellular signaling (Li et al., 2024; Nidhi et al., 2025). Emerging evidence indicates that membrane transporters not only underpin fundamental physiological and metabolic processes but also act as key regulatory hubs in stress tolerance mechanisms, particularly in modulating oxidative stress responses and conferring resistance to heavy metal toxicity (Akhiyarova et al., 2024; Mishra G. et al., 2024; Yuan et al., 2024; Faizan et al., 2024a). Moreover, their activity is finely modulated by complex phytohormonal signaling networks that integrate environmental cues with cellular responses. Plant hormones, or phytohormones, are central regulators of growth, development, and stress responses. Under abiotic or biotic stress conditions, hormones such as ABA, SA, JA, ET, auxins, cytokinins, and gibberellins coordinate complex signaling networks that modulate physiological, biochemical, and molecular responses. ABA, for instance, plays a pivotal role in drought and salinity tolerance by regulating stomatal closure, osmolyte accumulation, and expression of stress-responsive genes (Sultan et al., 2025). Similarly, JA and SA orchestrate defense responses against pathogens, while auxins and cytokinins adjust growth patterns to optimize resource allocation under stress. Hormonal crosstalk allows plants to fine-tune responses, integrating multiple stress signals and ensuring survival. The dynamic regulation of hormone biosynthesis, transport, and signaling enables plants to adapt rapidly to changing environments, making hormonal control essential for stress resilience and overall fitness.

Phytohormones exert multifaceted regulatory control over membrane transport systems by modulating the transcription of transporter genes, determining their subcellular targeting, and adjusting their post-translational activity. Through these integrated regulatory layers, plants achieve a dynamic balance that enables rapid adaptation to environmental fluctuations and developmental transitions (Lin et al., 2024; Mishra G. et al., 2024; Faizan et al., 2023). Among these hormonal regulators, abscisic acid (ABA) and auxin play central roles in orchestrating transporter-mediated ion fluxes, metabolite allocation, and signaling network coordination, thereby linking stress responses with developmental patterning (Akhiyarova et al., 2024; Mishra S. et al., 2024; Cui et al., 2023). Advancing our understanding of these intricate molecular mechanisms will be instrumental in strengthening plant resilience, optimizing nutrient and water use efficiency, and formulating targeted biotechnological interventions for sustainable crop improvement.

Although most plant cells possess the inherent ability to synthesize phytohormones due to their totipotency, the spatial and temporal precision of hormone transport largely dictates their biological functions (Mikuła et al., 2022). The concept of polar auxin transport, proposed more than a century ago, established the foundation for elucidating how directional hormone movement depends on asymmetrically localized transporter proteins such as the PIN-FORMED (PIN) efflux carriers (Hammes and Pedersen, 2024). Auxin has traditionally been considered the principal determinant of developmental polarity; however, emerging evidence reveals that other hormones, including strigolactones (SLs) and cytokinins (CKs), also exhibit directional transport mediated by specific transporter proteins. For instance, SLs can be selectively secreted into the rhizosphere, modulating root system architecture and facilitating interactions with symbiotic microorganisms (Sharma and Marhava, 2023).

**Figure 1:**
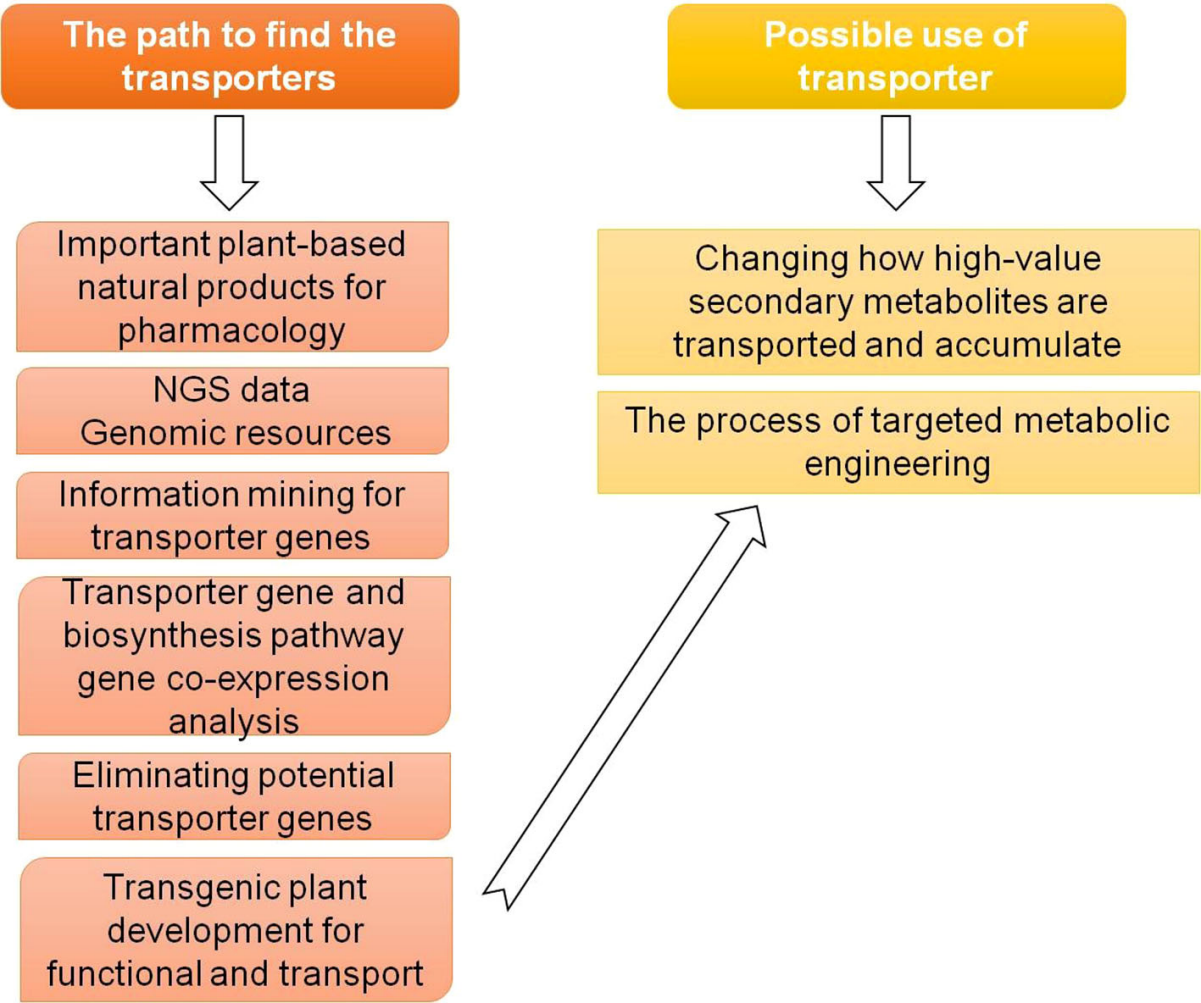
Schematic diagram showing the discovery and likely applications of the transporters.

Phytohormones exhibit both short- and long-distance mobility within plants, facilitated by multiple transport routes including cell-to-cell diffusion, apoplastic flow, and vascular transport through the xylem and phloem (Alam et al., 2025; Faraz et al., 2023). While long-distance translocation through vascular tissues predominantly relies on transpiration-driven bulk flow and hydrostatic pressure gradients, the selective loading and unloading of hormones are tightly regulated active processes governed by specific membrane transporters (Klíma et al., 2016). At the cellular level, phytohormone molecules may cross membranes passively, following concentration gradients, or actively, via specialized transporter systems that confer substrate specificity and spatial control. These transporters act as key modulators of hormone homeostasis and signal distribution, ensuring precise coordination between local signaling and systemic responses, as illustrated in **Figure 1**. Moreover, their activity is intricately regulated by various mechanisms, including transcriptional and translational control, post-translational modifications, and vesicular trafficking of transporter proteins to and from the plasma membrane (SenGupta et al., 2021).

Despite their structural conservation, membrane transporter families such as ATP-binding cassette (ABC), nitrate/peptide transporter family (NPF), and multidrug and toxic compound extrusion (MATE) proteins exhibit remarkable functional plasticity, accommodating an extensive array of substrates with diverse chemical properties (Léran et al., 2014; Longo et al., 2018). Initially characterized as nitrate transporters, NPF proteins have since been recognized as multifunctional carriers capable of mediating the translocation of a broad spectrum of phytohormones, including indole-3-acetic acid (IAA), indole-3-butyric acid (IBA), gibberellins (GAs), abscisic acid (ABA), jasmonic acid (JA), and jasmonate conjugates (Shimizu et al., 2021). For instance, NPF2.10 (GTR1) has been demonstrated to facilitate the transport of glucosinolates, gibberellins, and jasmonoyl-isoleucine (JA-Ile), reflecting its multifunctional substrate recognition capacity (Saito et al., 2015). Representative examples of such multifunctional transporters are summarized in **Table 1**. This exceptional substrate promiscuity underscores the evolutionary adaptation of plant transport systems, enabling the coordination of complex metabolic, hormonal, and signaling networks through a relatively limited repertoire of transporter proteins (Tal et al., 2016; Zhang Y. et al., 2023; Faizan et al., 2024b).

**Table 1:** Examples of role of membrane transporters in plants, highlighting their functions in nutrient uptake, name of transporter proteins and their location and localization in plants.

Plant species	Transporter protein	Localization of plants	Function(s)	Reference(s)
*Petunia hybrida*	Transporters of volatile compounds PhABCG1	Plasma membrane	Mediates the release of floral volatile compounds	Adebesin et al. (2017)
Strawberry	FaTT12-1	Tonoplast	Promotes the accumulation of proanthocyanidin.	Chen et al. (2018)
*Artemisia annua*	AaPDR3	Plasma membrane	Sesquiterpenes β-caryophyllene is transported	Fu et al. (2017)
*Crocus sativus*	CsABCC4	Tonoplast	Functions as a regulatory node in vacuolar transporter activity	Demurtas et al. (2019)
*Vitis vinifera*	VvMATE2	Golgi complex	The transporter of proanthocyanidin in the Golgi complex	Pérez-Díaz et al. (2014)
*Arabidopsis thaliana*	AtABCC2	Tonoplast	Mediates the movement of anthocyanins and other flavonoids through the vegetative tissues’ vacuoles	Behrens et al. (2019)
*Papaver sominferum*	BUP1	Plasma membrane	Transporter of benzoylisoquinoline alkaloids	Dastmalchi et al. (2019)
*Medicago truncatula*	MtABCG10	Plasma membrane	Controls flavonoid release, root isoflavonoids, and liquiritigenin/4-coumarate transport	Biała et al. (2017)
*Camellia japonica*	CjMATE1	Tonoplast	Promotes the accumulation of berberine in the vacuoles	Takanashi et al. (2017)
*Catharanthus roseus*	CrNPF2.4, CrNPF2.5, CrNPF2.6	Plasma membrane	Carries the iridoid glycosides organic acid, loganin, secologanin, and 7-deoxyloganic acid	Larsen et al. (2017)
*Triticum aestivum*	TaPTR2.1	Tonoplast	Controls the water status during the early stages of seed germination	Choi et al. (2020)
*Zea mays*	ZmSUT2	Tonoplast	Remobilizes vacuolar sucrose to support tissue development via H^+^-coupled transport	Leach et al. (2017)
*Brassica napus*	BnaPHT1	PM	BnaPHT1 regulates Pi acquisition, homeostasis, and multi-nutrient stress responses in *B. napus*.	Li et al. (2019)
*B. napus*	BnaPHT1;4	Cotyledons of early developing seedlings	Pi homeostasis, ABA and GA biosynthesis modification, seed germination, and seedling growth	Huang et al. (2019)

Because heterologous systems like yeast or oocytes can only partially recreate the *in-vivo* environment, confirming transporter function requires complementary approaches. Phenotypic analyses of transporter mutants, hormone rescue experiments, hormone quantification, and fluorescent tracer studies in plants provide critical validation of transporter roles (Rottmann et al., 2018; Parker et al., 2017; Zhang et al., 2021). Ultimately, the evolution of multiple-substrate transporters likely reflects an adaptive strategy to balance metabolic efficiency and signaling precision (Zhang N. et al., 2023).

In this review, we present a comprehensive and integrative overview of the intricate regulatory interplay between phytohormones and membrane transporters in plants. Specifically, we elucidate how diverse classes of phytohormones including auxins, abscisic acid (ABA), cytokinins (CKs), gibberellins (GAs), ethylene, jasmonates (JAs), salicylic acid (SA), and strigolactones (SLs) govern the transcriptional regulation, subcellular localization, and functional modulation of membrane transport proteins. We further explore the molecular and signaling mechanisms through which phytohormone-mediated control of transporter activity orchestrates nutrient acquisition, ion homeostasis, metabolite partitioning, and detoxification processes. By integrating current advances from molecular biology, genomics, and physiology, this review also highlights how transporter–hormone crosstalk underpins key aspects of plant development and stress adaptation, including responses to abiotic and biotic stressors. Ultimately, we aim to provide a unified perspective on how phytohormonal regulation of membrane transport serves as a central hub connecting environmental sensing with metabolic and developmental reprogramming offering novel insights for improving crop productivity and resilience through targeted genetics and biotechnological interventions.

## 2 Phytohormones and molecular mechanisms of membrane transporters signaling cascade in plants

**Figure 2:**
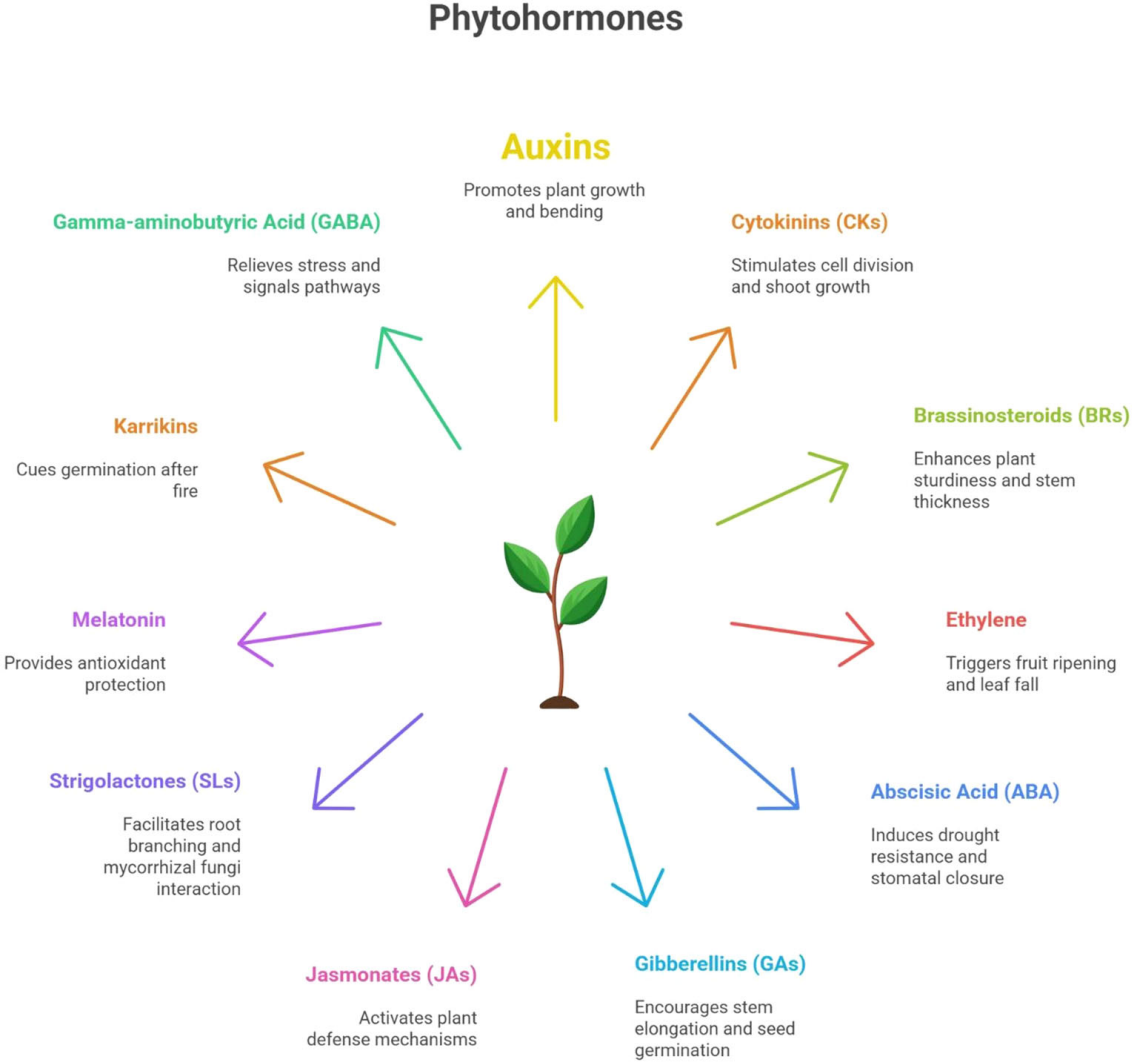
Schematic diagram presenting types of phytohormones discussed in this review and their major functions in the plants.

The response of plant tissues to PGRs is strongly influenced by both the relative proportions of different hormone classes and the specific concentrations of individual growth regulators. The expression of genes governing hormone homeostasis is subject to intricate regulatory interactions, where one hormone may either upregulate or repress the biosynthesis, transport, or signaling of another. This inter-hormonal crosstalk creates a complex and dynamic regulatory network modulated by multiple internal and external factors. Furthermore, PGRs can exert synergistic or antagonistic effects on the same set of genes or proteins, adding an additional layer of complexity to their mechanistic understanding (Kaya et al., 2023). As dynamic organisms, plants continuously perceive and adapt to environmental fluctuations. To coordinate growth, development, and stress responses, they rely on a suite of small organic signaling molecules known as plant hormones or phytohormones (**Figure 2**), which function through intricate multi-level interactions, which fine-tune numerous physiological and molecular processes (Aizaz et al., 2024).

**Figure 3:**
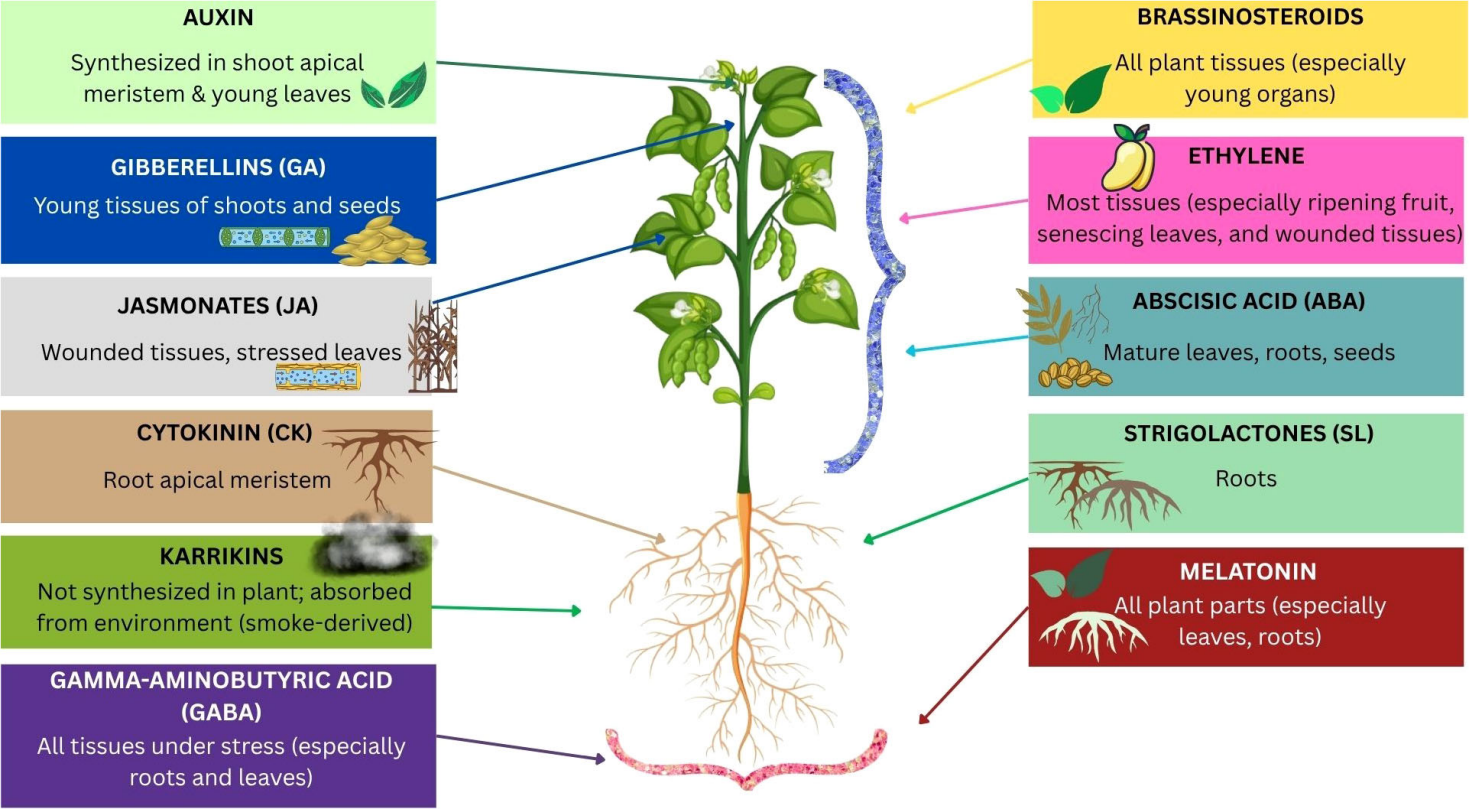
This figure illustrates different types of phytohormones and their primary synthesis sites in a plant. Arrows and icons pinpoint the specific regions, such as shoot tips, roots, and leaves, where each hormone is produced.

**Figure 4:**
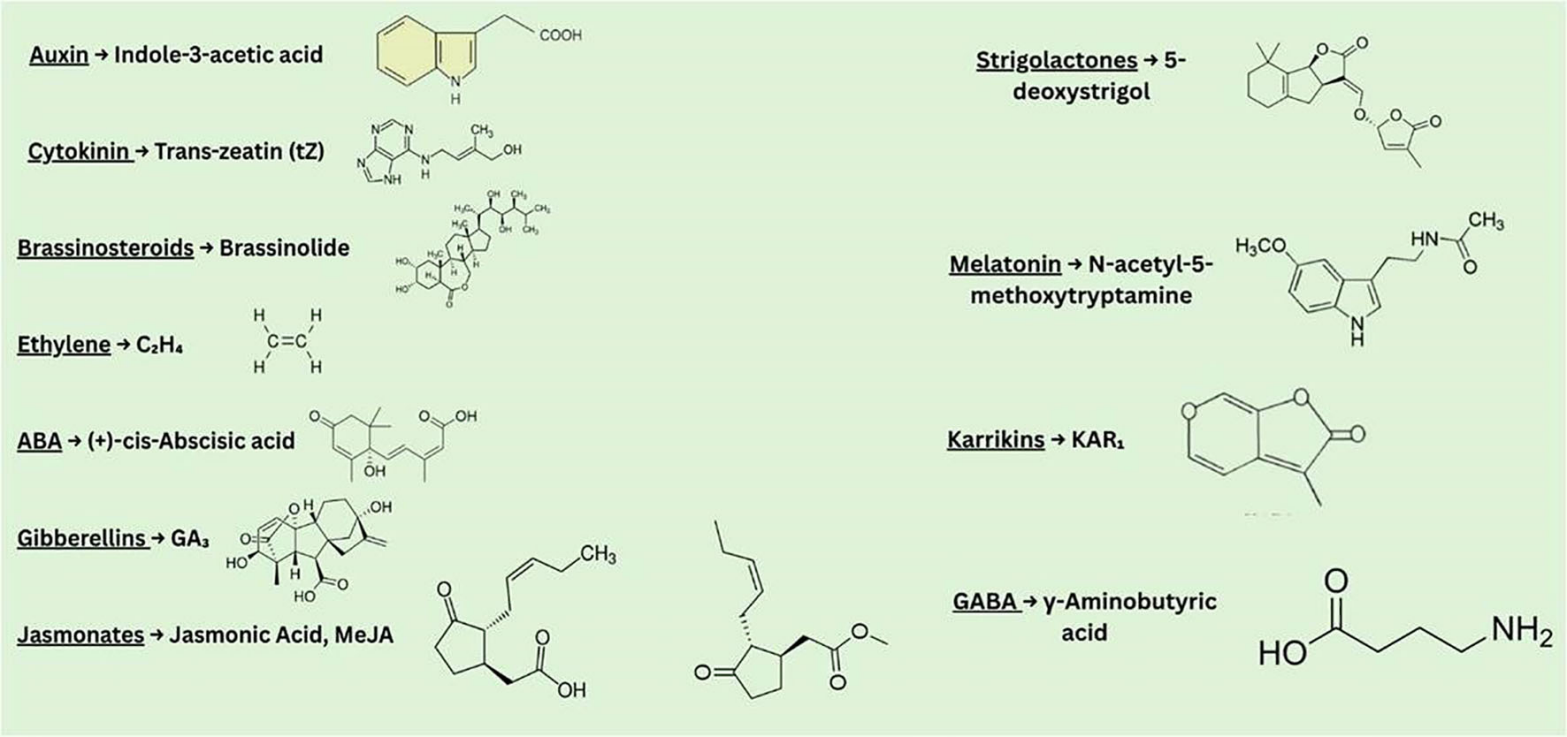
This diagram represents the bioactive compound forms of various phytohormones and their chemical structures.

Hormone synthesis, transport, perception, metabolism, and signal transduction all contribute to the overall regulation of hormone homeostasis (Sami et al., 2018; Faizan et al., 2020; 2024c). Perception of hormones may occur nearby or far away from the location of synthesis. Therefore, in order to control their distribution and trigger different reactions, active transporters can carry hormones to their site of action. Bioactive hormone forms exhibit this spatial regulation, which also holds true for their conjugated and intermediate forms (Wong et al., 2023). Each family of hormone transporters has distinct characteristics. Additionally, plants also translocate other mobile signaling factors like hormone-like molecules and small peptides (Zhang et al., 2025). **Figures 3** and **4** represents the schematic diagrams for biosynthesis locations for different types of phytohormones and their bioactive forms along with chemical structures. These mechanisms are also described in this following section.

### 2.1 Auxins

**Figure 5:**
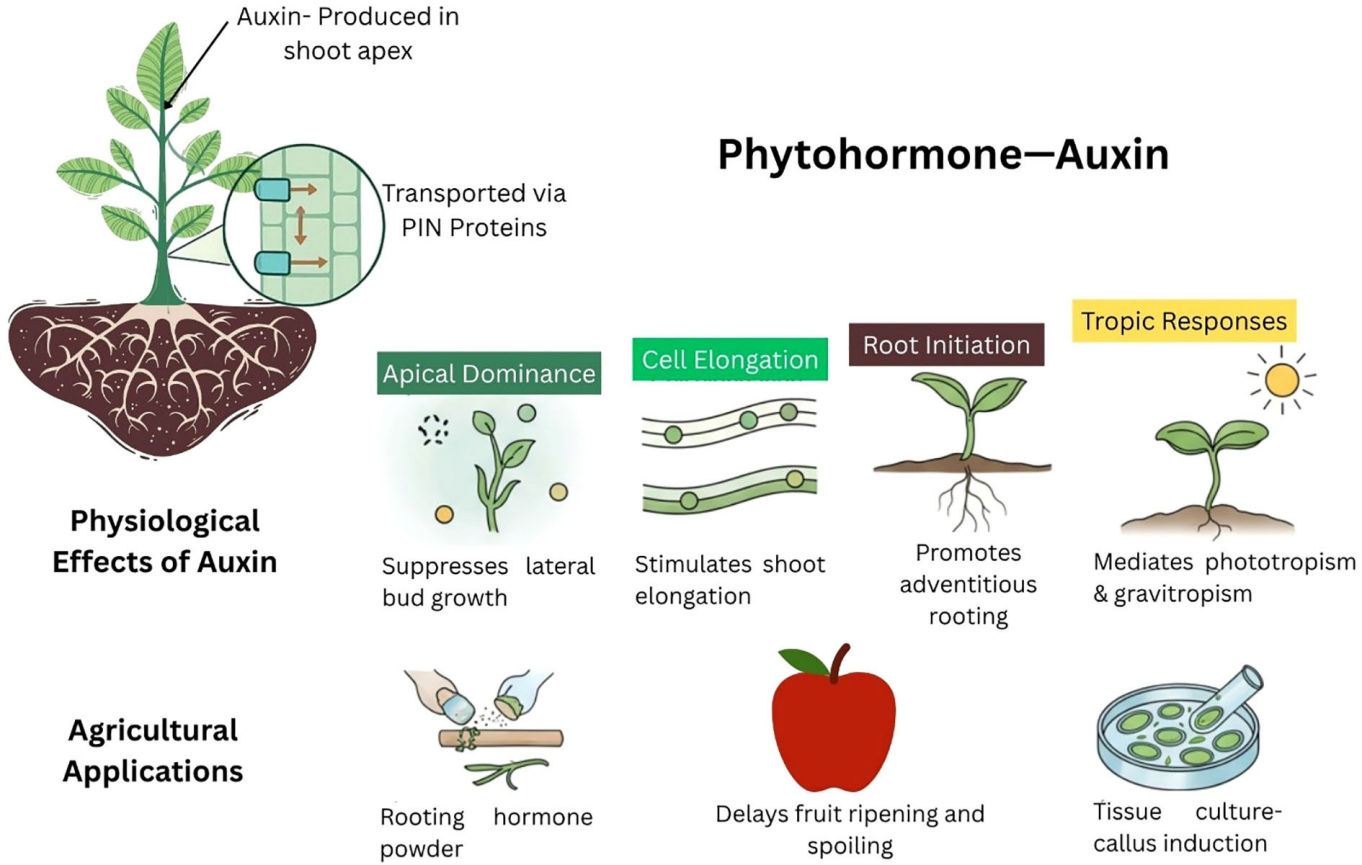
Diagram presenting details of phytohormones auxin, production location, physiological effects on plants and applications.

From the embryonic stage to senescence, auxins, primarily IAA controls a variety of aspects of plant growth and development. **Figure 5** shows the biosynthesis location, transport, physiological effects on plants and applications of auxins. Plant growth and development are significantly influenced by auxin, IAA, which regulates cell division, elongation, and differentiation. In *Arabidopsis*, peas, and rice, IAA promotes GA synthesis by deactivating GA2ox and activating GA3ox and GA20ox (Yin et al., 2007). It appears that tissue-specific responses, like those found in pea plant roots, are where auxins have a positive impact on GA content (Weston et al., 2009). Conversely, GAs regulates genes associated with auxin. However, the effects depend on the specific combination of ARFs induced by GAs. For instance, GA_3_ negatively regulates hypocotyl elongation. In contrast, ARF6 and ARF8 promote it (Zhang et al., 2019). GAs also alters the expression of auxin transporters. In Arabidopsis, PIN proteins (PIN1, PIN2, and PIN3) show reduced activity in signaling-deficient mutants and GA biosynthesis mutants. GA treatment restores the wild-type phenotype, indicating that GAs are essential for proper PIN protein function (Willige et al., 2011). The biological implications of this GA-dependent regulation include the promotion of xylogenesis in *Populus* through PIN1 up-regulation and the modulation of gravitropism in *Arabidopsis* roots through PIN2 stabilization.

Exogenous GA3 treatment in *Eucalyptus* roots and stems stimulates xylogenesis and modifies the expression of genes linked to auxin, secondary cell wall formation, and GA biosynthesis. As found in some studies, under IBA and GA3 treatment, hypocotyl sections of *Pinus radiata* seedlings also exhibit xylem differentiation. The fact that GAs also influences auxin content and signaling further complicates their relationship (Liu et al., 2018). The relationship between auxins and GAs involves multiple levels, often in a tissue-specific manner, including signaling, metabolism, or gene expression. Both hormones appear to have a synergistic nature, as they positively impact certain aspects of development, even though a clear relationship between them cannot be established (Devesa and Caicedo, 2019).

Transport mechanism: Local hormone maxima are produced when auxin input and output transporter proteins work together. Anfang and Shani (2021) assert that basic developmental processes like organ development, light bias (phototropism), and directional root growth (hydrotropism and gravitropism) depend on directional auxin gradients. The most common bioactive form of plant auxin is IAA (**Figure 4**). Auxin is transported by a variety of pathways, such as plasmodesmata, vasculature systems, transporter-mediated directional cell-to-cell polar auxin transport, non-directional passive movement, and other non-polar transport-related processes (Mazzoni-Putman et al., 2021).

Auxin transporters are divided into several groups. Among them, PIN proteins are well-characterized. They facilitate polar IAA movement from cell to cell (**Figure 5**). In Arabidopsis, the eight PIN proteins differ in the length of their central hydrophilic loops (Geisler, 2021). The long PIN proteins (PIN1–4 and PIN7) have polar localization, which directs auxin efflux. These proteins are located in the plasma membrane and act as exporters. In contrast, PIN5, PIN6, and PIN8 have shorter central hydrophilic domains. PIN6 is found in both the plasma membrane (PM) and the endoplasmic reticulum (ER), while PIN5 and PIN8 are restricted to the ER. This suggests that these proteins may regulate intracellular auxin homeostasis.

**Figure 6:**
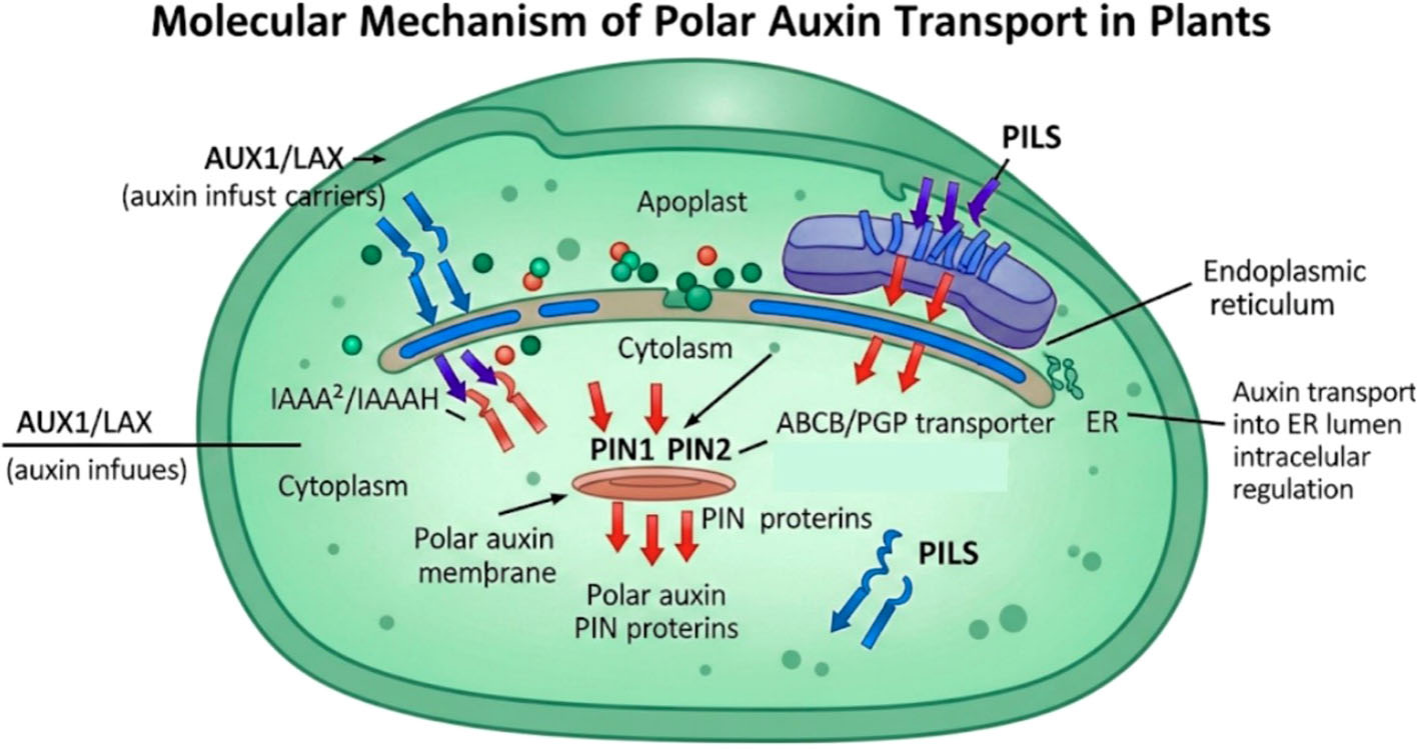
A schematic diagram presenting molecular mechanism of polar auxin transport in plant cells. Auxin in the form of IAA enters the cell via passive diffusion or through active influx carriers (AUX1/LAX family) at the apical membrane. Once in the cytoplasm, auxin is actively exported by PIN efflux carriers localized basally, establishing directional/polar transport. ABCB/PGP transporters aid in long-distance auxin flow and interact with PIN proteins for transport stabilization. Intracellularly, PILS proteins on the ER membrane regulate auxin homeostasis by controlling subcellular sequestration. This coordinated action of transporters establishes auxin gradients critical for plant development and tropic responses.

At a particular auxin binding site, each monomer in the homodimer structure is separated into transport and scaffold domains. Changes in structure related to transport are made possible by a proline-proline transition. Therefore, transport activity is likely driven by auxin’s negative charge and is not affected by proton and ion gradients (Luschnig and Friml, 2024). PIN phosphorylation should be taken into consideration in auxin transport studies since recent research has shown that it is necessary for PIN activation (Seifu et al., 2024). Additionally, the central hydrophilic loop and the N- and C-terminal transmembrane domains are implicated in cellular polarity and subcellular localization, as evidenced by the systematic domain alternation between PIN proteins localized to the PM and ER and PINs localized to the apical and basal plasma membrane (**Figure 6**). By establishing differential growth rates through auxin sequestration, the PIN-like PILS transporter family, found in the ER, appears to limit the nuclear auxin response (Bilanovičová et al., 2022). The growth of organs is negatively regulated by PILS6, and its abundance rises with temperature. Increased nuclear auxin signaling and root growth are caused by temperature-sensitive suppression of PILS6, which controls the nuclear availability of auxin.

In different tissues and organs, auxin-related developmental programs are mediated by the 4 functional auxin efflux transporters that comprise the AUX/LAX family. To control root gravitropism and root hair development, the most researched family member, AUX1, needs auxin transport from the root tip to the shoot direction. AUX1 has been shown to promote root hair elongation in rice and *Arabidopsis* in response to phosphate limitation (Ng et al., 2015). When combined with cell type-specific auxin biosynthesis induction, single-cell nucleus morphogenetic tracking can be used to map the direction of auxin flow in the root. According to experiments, auxin flows down the root in a manner that is PIN-dependent but AUX1/LAX-independent. Auxin movement from the epidermis to the vasculature produces similar outcomes. AUX1 and PIN2 are required for auxin to move efficiently into the shoot. Local auxin concentrations are also essential for root bending. However, PIN2 and AUX1 likely do not control this process (Hu et al., 2021). A recent study showed that ABCB15–18 and ABCB22 regulate lateral root initiation. They do so by transporting IAA from the lateral root cap through the epidermis and into the shoot twice (Chen et al., 2019).

IAA has been shown to be exported from the vacuole to the cytosol by the auxin transporter WAT1, which is present in tonoplast. Additionally, several NPF proteins transport auxins. It is the dual affinity nitrate transporter NRT1.1 that transports IAA and mediates nitrogen uptake from the rhizosphere (Krouk et al., 2010). NPF transporters are known to exist for indole-3-butyric acid (IBA), the precursor of IAA. The sequestration of IBA into the vacuole is encouraged by the first of these, TOB1 (also referred to as NPF5.12); the second, NPF7.3, controls gravitropic responses by delivering IBA into columella stem cap cells (Anfang and Shani, 2021). Together with these two NPF IBA transporters, other proteins involved in IBA transport include PXA1 (also called ABCD1) (Zolman et al., 2001), ABCG36 (Strader and Bartel, 2009), and ABCG37. IBA uptake transporters and long-distance transport efflux transporters, however, are not known to be regulated (Damodaran and Strader, 2019).

The close coordination between auxin and GA forms a complex regulatory network influencing diverse developmental processes, including elongation, xylogenesis, and gravitropism. Auxin not only promotes GA biosynthesis but also modulates GA-responsive genes and transporters, highlighting their synergistic nature. The coordinated activity of auxin transporters such as PIN, AUX/LAX, PILS, ABCB, and NPF proteins ensures precise spatial distribution and signaling. Together, these mechanisms maintain hormonal balance and enable plants to adapt dynamically to developmental and environmental cues.

### 2.2 Cytokinins

**Figure 7:**
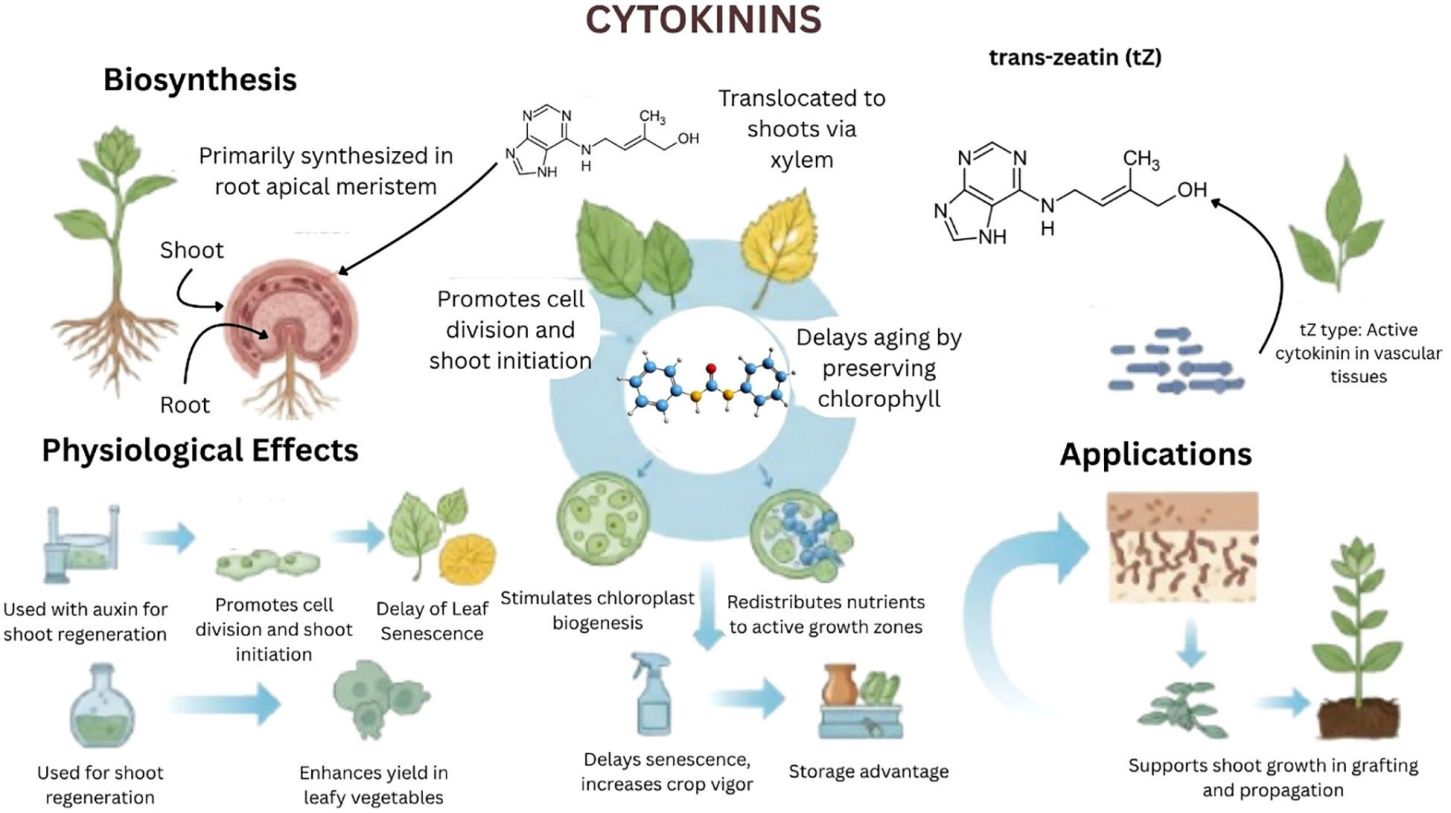
Key functions and agricultural applications of cytokinins, particularly the trans-zeatin (tZ)-type. Cytokinins regulate shoot growth, delay senescence, and enhance nutrient mobilization. Their role in tissue culture and postharvest preservation makes them valuable tools in modern agriculture and plant biotechnology. Grafting studies help clarify ABCG14’s role in transporting CKs from root to shoot. Wild-type rootstocks can restore growth in ABCG14 mutant scions. In contrast, grafting wild-type scions onto ABCG14 rootstocks results in growth defects similar to ABCG14 mutants (Ko et al., 2014). Transporters for iP-type CKs have not yet been identified, and their basipetal transport is not well understood. CKs and GAs work together to regulate morphological and physiological traits in Polygonum cuspidatum in response to nitrogen availability. Sugiura et al. (2015) reported that endogenous CK and GA levels increase under high nitrogen, indicating that both hormones contribute to biomass allocation.

Numerous biological processes in plants are linked to CKs, such as a class of mobile adenine derivatives, nutrient homeostasis, apical dominance, leaf senescence, root nodulation, and cell division and differentiation as show in **Figure 7** (Werner and Schmülling, 2009). For instance, in order to control shoot development, nitrate causes roots to produce more trans-zeatin (tZ)-type CKs, which are subsequently carried to shoots by the xylem (Ruffel et al., 2016). On the other hand, to control nitrogen uptake, root nodulation, and preserve vascular order within the root meristem, N6-(D2-isopentenyl) adenine (iP)-type CKs have been observed to migrate from shoots to roots *via* phloem (Kiba and Krapp, 2016). The ability of both root-derived and shoot-derived CKs to travel acropetally and basipetally through the plant vascular system is demonstrated by chemical profiling related to grafting experiments between CK biosynthesis mutants. Although prior *in-vitro* research has suggested that the purine permease (PUP) and stabilizing nucleoside transporter (ENT) families mediate CK translocation at the molecular level, their function in long-distance CK transport in plants is unknown (Kiba et al., 2013). According to studies conducted on *Arabidopsis*, ABCG14, a G-type ABC transporter, is necessary for the long-distance transport of tZ-type CKs from roots to shoots (Ko et al., 2014). In the root differentiation zone, stem cells that overlap with CK production sites express ABCG14 which is found in the plasma membrane at the subcellular level and most likely serves as an efflux transporter.

Transport mechanism: The maintenance of shoot and cambial meristem activities is one of the many physiological and developmental processes that CKs support in addition to cell division, differentiation, and root nodulation (Werner and Schmülling, 2009). The long-distance transport of root-derived CK in rice is regulated by OsABCG18, indicating that it shares characteristics with ABCG14 in *Arabidopsis*. According to Zhao et al. (2019), members of the ENT family have also been proposed as mediating CK transport. PUP14 transfers CK from the apoplast to the cytosol (*Arabidopsis*), reducing the amount of bioactive CK in the apoplast and the CK response (Zürcher et al., 2016). The PUP family includes other members who are categorized as rice CK importers. CK is transported from the apoplast to the cytosol by OsPUP4, while it is imported into the ER by OsPUP1 and OsPUP7. These transporters work together to mediate CK transport, which regulates rice grain size and development (Xiao et al., 2020).

**Figure 8:**
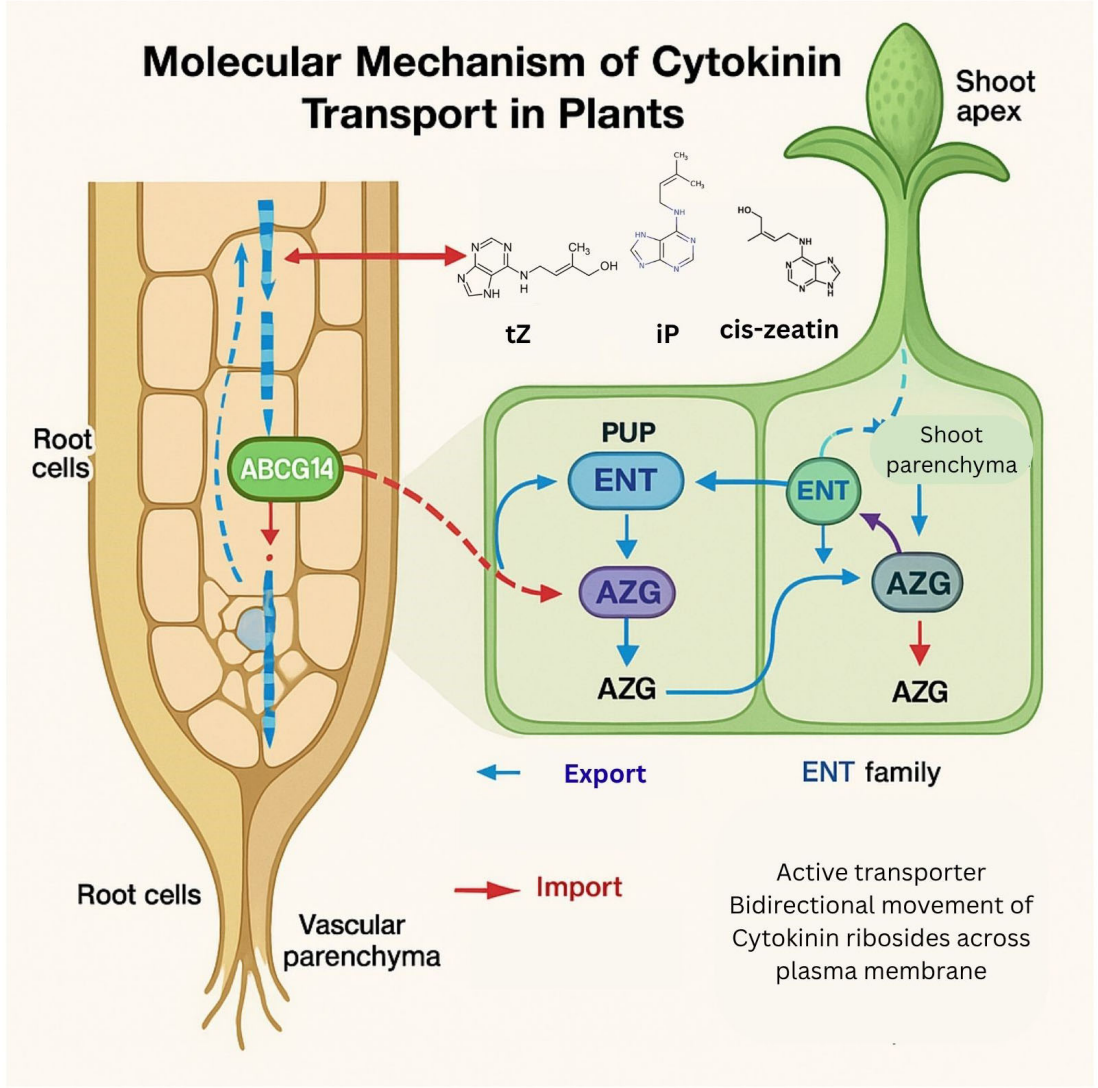
Schematic representation of molecular mechanism of cytokinin transport in plants. This schematic illustrates the synthesis, cellular localization, and transport routes of CKs, primarily trans-zeatin (tZ), from root tissues to shoot meristems. Key transporters such as ABCG14 mediate long-distance translocation via the xylem, while PUP, ENT, and AZG transporters facilitate local uptake and distribution across cellular membranes.

In *Arabidopsis*, CK transporters known as AZA-GUANINE RESISTANT (AZG) purine transporters contribute to the interaction between auxin and CK. AZG1, a CK importer, directly interacts with PIN1 and co-localizes when roots are under stress. Furthermore, it has been suggested that ABCI19, ABCI20, and ABCI21, all of which belong to the ABCI-type transporter family, coordinate to function as CK transporters (**Figure 8**). It has been proposed that these ER-localized proteins lower cytosolic CK levels. The xylem’s CK is thought to be a mobile signal that causes nearby pro-cambium cells to divide (Tessi et al., 2023).

Kinetin was the first CK to be isolated from maize endosperm in 1955, before trans-Zeatin (tZ) was isolated in 1961. To restore the shoot growth phenotype, tZ is transported acropetally to the shoot in mutants ATIPT1, ATIPT 3, ATIPT5, and ATIPT7, but not in iP, according to grafting experiments. On the other hand, iP is sufficient for normal root function and commands transport in the basipetal direction. The transport of CKs over long distances is an essential step in their regulation of plant growth and development. For instance, iP-type CKs derived from shoots control nodulation in *Lotus japonicas* roots and vascular patterning in the *Arabidopsis* root meristem, whereas tZ-type cytokinins derived from roots stimulate shoot growth (Dean et al., 2022). But in the second case, the authors found that the gene AtABCG14 is highly co-expressed with the biosynthesis of cytokines, suggesting that it plays a role in CK transport. AtABCG14 mutants exhibited shorter and thinner flower stalks and significantly smaller rosette leaves compared to the wild type. Additionally, the mutant’s xylem and phloem cell count and sizes were significantly lower. In plants where the AtABCG14 gene had been knocked out, tZ spraying restored the phenotype, confirming the hypothesis that AtABCG14 might facilitate the movement of tZ-type cytokinins from root to shoot. Research indicates that the appropriate nodule establishment and root growth of nitrogen-fixing plants depend on this transport mechanism (Ko et al., 2014).

These findings illustrate how cytokinins function as key mobile signals integrating root-shoot communication and coordinating growth, development, and nutrient responses. Their long-distance transport, primarily mediated by ABCG14/OsABCG18 for trans-zeatin and by PUP, ENT, and AZG transporters for iP-type CKs, ensures precise hormonal distribution. The differential movement of tZ and iP forms maintains the balance between shoot growth and root development. Together, these mechanisms highlight the sophisticated regulation of CK transport essential for plant homeostasis and adaptive growth.

### 2.3 Brassinosteroids

The TF BRASSINAZOLE RESISTANT 1 (BZR1), which controls BR responses in plants, is activated by GAs following DELLA degradation. Similarly, BRs influence how plants react to light by boosting the transcriptional activity of PITOCROME INTERACTING FACTORS (PIF) TFs (Zhang et al., 2019). This is the intersection of BRs and GAs, given that GAs allows PIF4 activity, which in turn regulates the expression of GA3ox and GA20ox (Ferrero et al., 2019). Unterholzner et al. (2015) claim that, in addition to increasing the expression of BZR1 and BRI1 EMS SUPPRESSOR 1 (BES1), overexpression of the BR biosynthesis genes DWARF 4 (DWF4) and GA20ox also increases GA levels. According to de Vleesschauwer et al. (2012), certain oomycete suppress rice’s immune responses by inhibiting GA biosynthesis through elevated BR levels. Although some studies support and give molecular mechanisms by which BRs and GAs reciprocally control each other’s activities, this connection is discovered lacking in peas, sunflower, and *Arabidopsis*, suggesting that these associations are conditioned only for particular phases or species.

Transport mechanism

Plant development is controlled by steroid hormones called BRs (Fujioka and Yokota, 2003). However, it is unclear whether and how BRs are transported throughout the plant. In Pisum sativum, BR accumulation was observed in various tissues, but there was no evidence of long-distance transport (Symons and Reid, 2004). BR biosynthesis enzymes are located in the ER, while BR receptors are on the cell surface (Fujioka and Yokota, 2003). Therefore, BR must be transported from the intracellular space to the apoplast, either passively or actively. Recent studies show that castasterone, brassinolide, and BR precursors can move locally over short distances within the root. This was revealed through cell-type misexpression studies and mapping of BR biosynthesis enzyme expression. Along the root axis, hormone gradients are formed by controlled biosynthesis and movement. Identifying BR export activity and specific carriers for BR and its precursors remains critical (Vukasinovic and Russinova, 2018).

**Figure 9:**
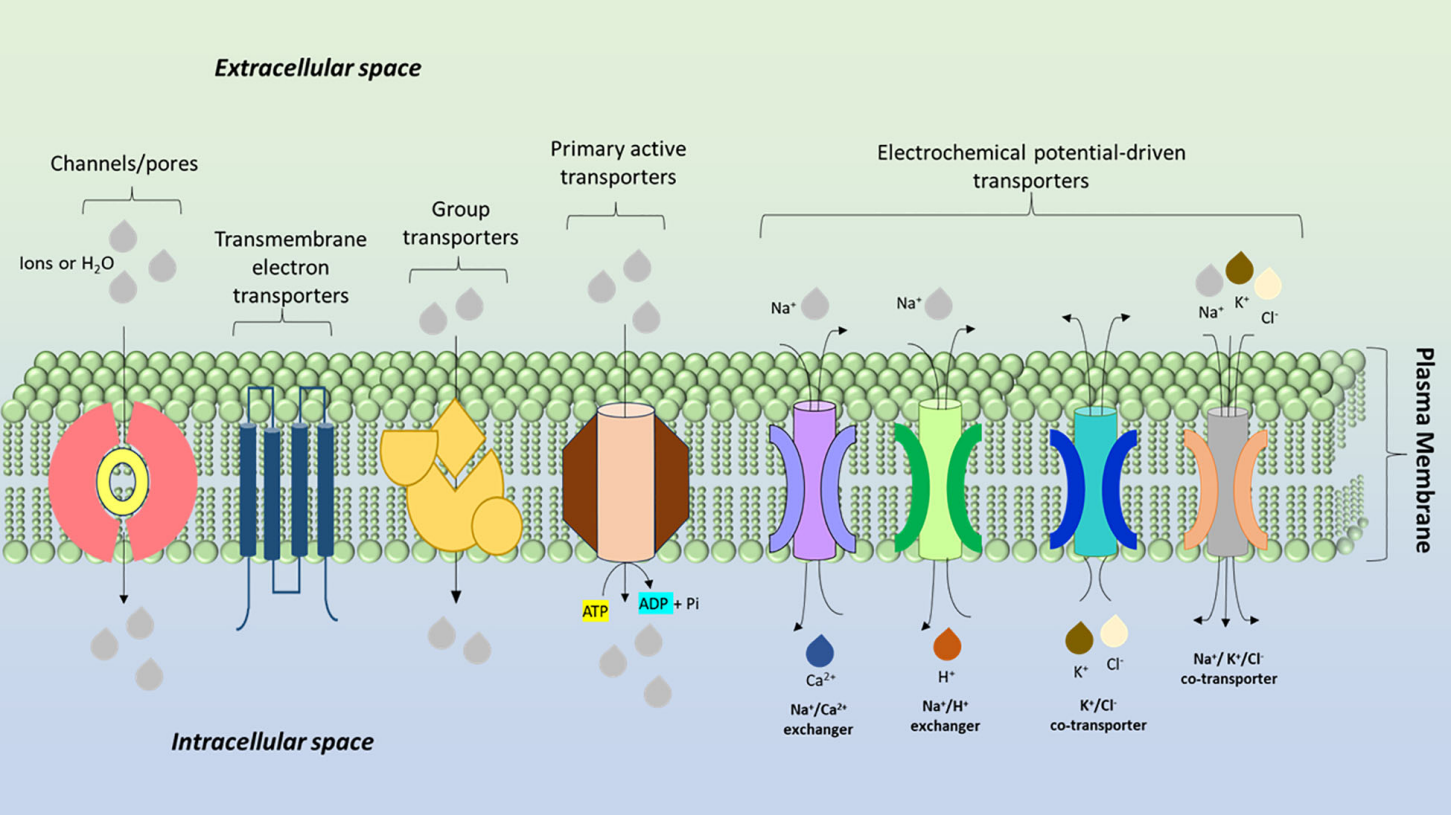
Types of membrane transport proteins in the plasma membrane of a plant cell: The diagram illustrates the different classes of membrane transport proteins involved in the movement of ions and molecules across the plasma membrane in plant cells.

**Figure 10:**
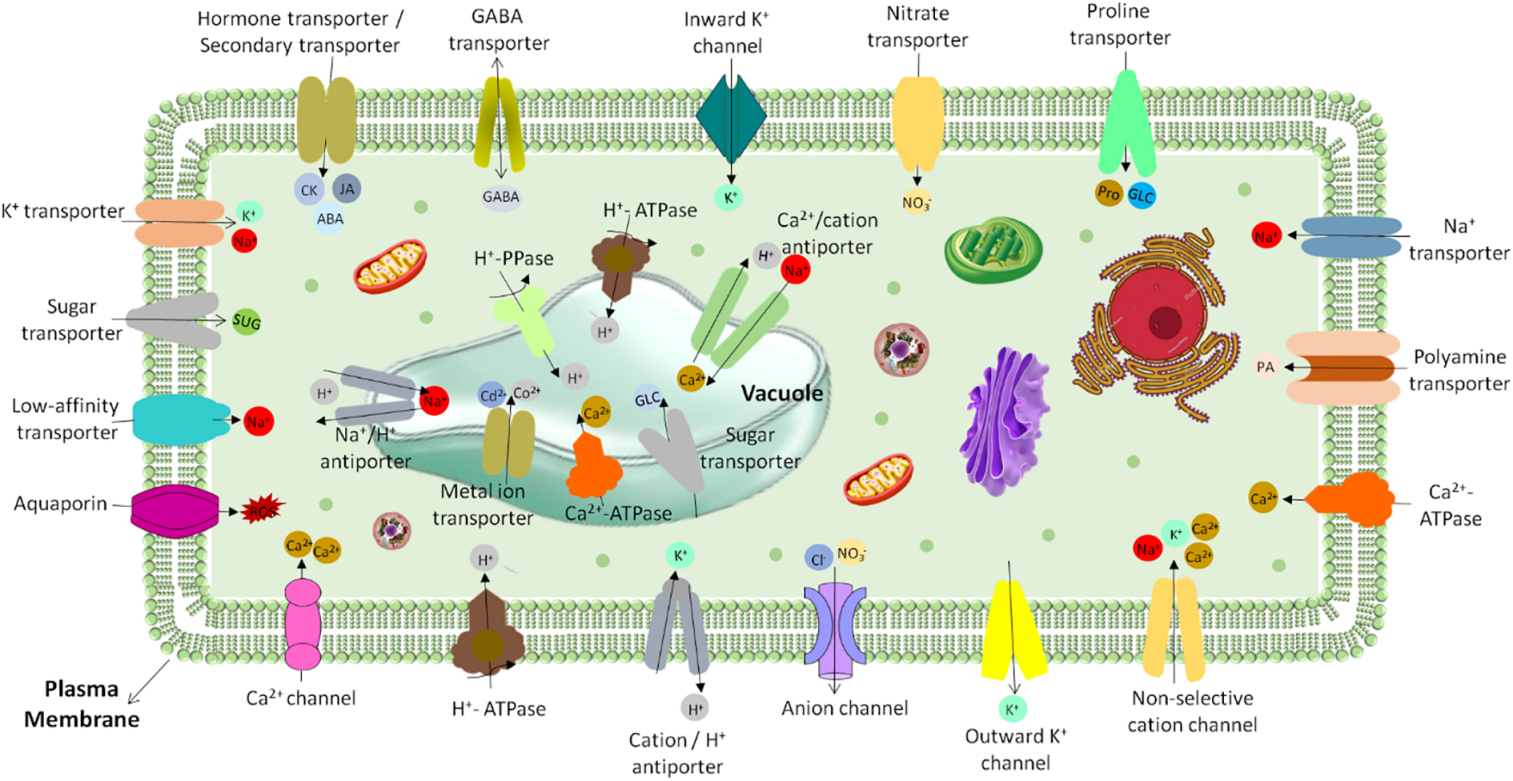
Transporters and channels in plant cell membranes and vacuoles: The diagram shows various membrane transport proteins localized on the plasma membrane and tonoplast (vacuolar membrane) of a plant cell. These transporters facilitate the uptake, compartmentalization, and efflux of essential ions, nutrients, and signaling molecules. The diagram highlights the complex regulation of ion homeostasis, nutrient distribution, and signaling in response to the plant’s physiological and environmental needs.

**Figure 11:**
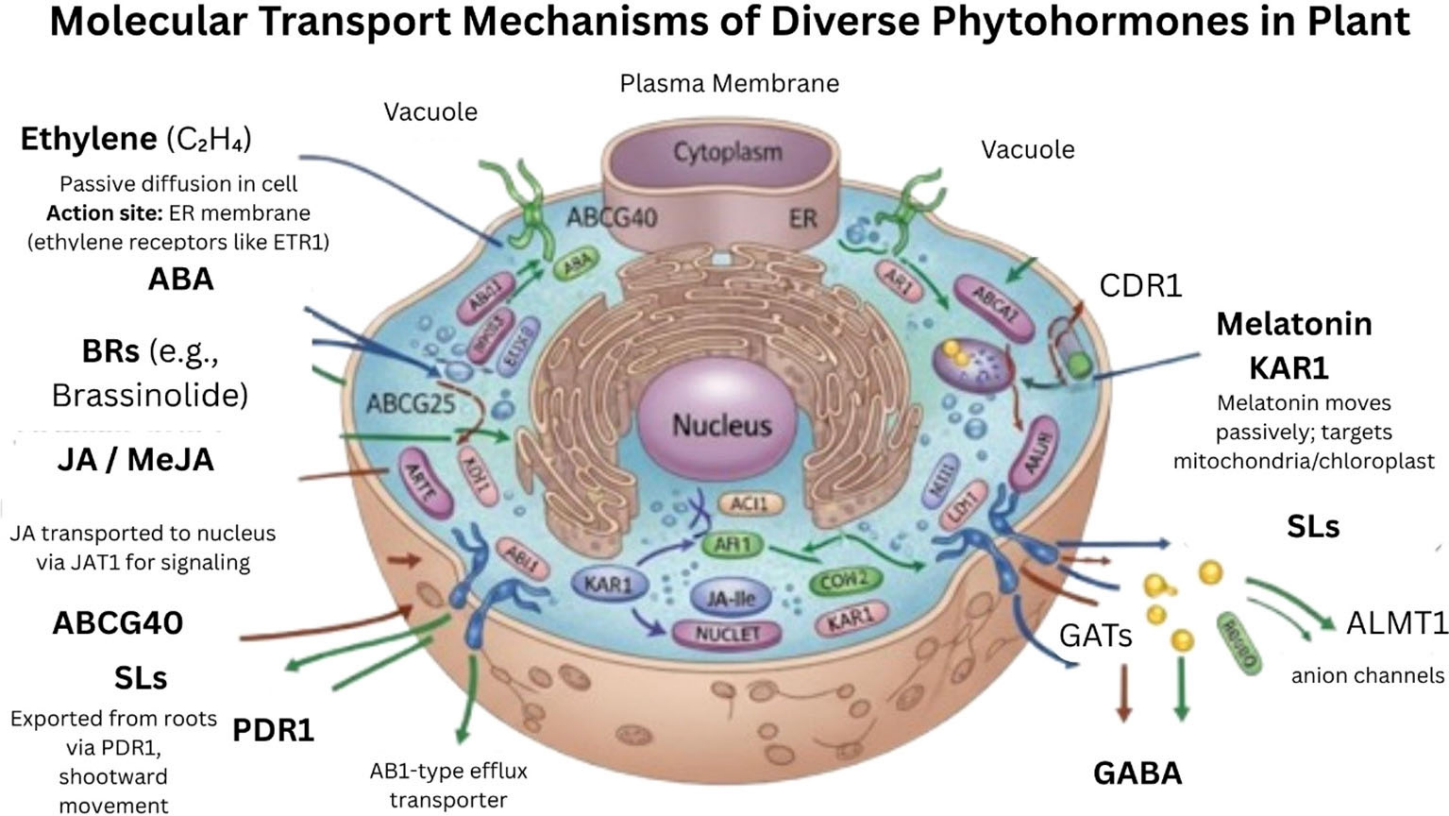
Schematic representation molecular transport mechanisms of selected phytohormones in plant cells. The schematic illustrates the intracellular and intercellular movement of ethylene, abscisic acid (ABA), brassinosteroids (BR), jasmonic acid (JA), melatonin, karrikins, strigolactones (SL), and gamma-aminobutyric acid (GABA) through passive diffusion, specific membrane transporters (e.g., ABC transporters, JAT1, PDR1, GATs), and recognition by key receptors (e.g., ETR1, BRI1, COI1, KAI2). Transport routes across the plasma membrane, endoplasmic reticulum, and into target organelles are depicted to highlight hormone-specific mobility and signal initiation sites.

BRs signaling increases nuclear abundance and improves auxin-mediated signaling by transcriptionally and post-translationally suppressing the accumulation of PILS proteins in the ER, according to a study (Luschnig and Friml, 2024) (**Figures 9**-**11**). ABCD1 (also called CTS), a member of the ABCD subfamily, imports IBA into peroxisomes. Based on an investigation, it is possible to map the directional auxin flow in the root and fine-tune the roles of PIN2 and AUX1 in this process by combining cell type-specific auxin biosynthesis induction with single-cell nucleus morphokinetic tracking (Hu et al., 2021). Additionally, it demonstrates how auxin transport depends on diffusion via plasmodesmata, which influences the distribution of auxin and encourages the growth and development of plant (Mellor et al., 2020). Lateral root emergence, phototropism, auxin distribution, and leaf hyponasty are all regulated by callose levels, which also affect auxin diffusion through the plasmodesmata (Mellor et al., 2020).BRs and GAs interact intricately to regulate plant growth and environmental responses through shared signaling components such as BZR1, BES1, and PIFs. While BRs enhance GA biosynthesis and signaling, the extent of this crosstalk varies across species and developmental stages. Despite evidence of localized BR movement, long-distance transport mechanisms remain unclear, emphasizing the need to identify specific BR carriers. Overall, the BR-GA interaction exemplifies the coordinated hormonal control underlying plant growth and adaptive plasticity.

### 2.4 Ethylene

Ethylene, a gaseous hormone, slows root development and has the capacity to readily diffuse within cells as well as traverse long distances across aerenchyma or vast intercellular gaps. GAs promotes primary root development, whereas ethylene inhibits it. As a result, it provides as an example of their distinctly opposing impacts on development. The capacity of ethylene to regulate GA homeostasis might explain this conflicting tendency. Studies have revealed that ethylene adversely regulates or modifies the genes involved in GA production in *Arabidopsis* (De Grauwe et al., 2008). The ethylene precursor 1-aminocyclo-propane-1-carboxylic acid (ACC) can move from root to shoot through the xylem when roots are subjected to waterlogging or hypoxia. Since ACC is an amino acid that is not found in proteins, the role of amino acid transporters in ACC transport has been hypothesized that the transporter is encoded by the lysine-histin transporter (LHT1) (Vanderstraeten et al., 2019). Wang et al. (2024) suggested that both the catabolic and biosynthetic GA genes may be regulated by ethylene. The interaction between GAs and ethylene in *Arabidopsis* seems to be essential for the apical hook’s development. GAs’ stimulation of ETHYLENE INSENTITIVE 3/EIN3-LIKE 1 (EIN3/EIL1), which exhibits light and close contact, enhances HOOKLESS1 (HLS1) expression after DELLA disintegration. This regulates the development of apical hooks and prevents their premature opening in etiolated seedlings (Zhao et al., 2021). The biosynthesis of ethylene is facilitated by activating ACC SYNTHASE5/ETO2 (ACS5/ETO2) and ACC SYNTHASE 8 (ACS8) as studied by Gallego-Bartolomé et al. (2011). In addition to expanding the apical hook, it inhibits their own action (Marín-de La Rosa et al., 2014). GAs and ethylene seem to have a complicated relationship overall, and depending on the particular process being studied, they may have opposing characteristics.

Transport mechanism: The lipid and aqueous environments of the cell allow the hormone ethylene to freely diffuse as a gas. Numerous aspects of plant development and environmental responses, such as seedling growth, organ development, abscission, maturation, and pathogen responses, are influenced by ethylene. A large amount of the spatiotemporal regulation of the gaseous hormone appears to be controlled by the location of its non-gaseous immediate precursor ACC, despite the fact that it can diffuse freely across membranes and travel between cells and the intracellular space without the assistance of transporters. ACC transport through the phloem has also been observed, despite the fact that the xylem is most likely to mediate the primary transport pathway of soluble ACC (Pattyn et al., 2021) (**Figures 9**-**11**).

Ethylene is a gas found in plants that diffuses quickly through lipid membranes and within cells. Ethylene lacks direct transporters, but the transportation of ACC, an ethylene precursor, is controlled (Tipu and Sherif, 2024). Diffusion is not possible for ACC since it is not a gas. Although the *Arabidopsis* ACC-resistant2 (are2) mutation reacts to ethylene normally, it exhibits a dose-dependent resistance to exogenously apply ACC. The amino acid transporter LHT1 is encoded by ARE2. AtLHT1 complements ACC insensitivity, and AtLHT2, a second ACC transporter, is discovered (Choi et al., 2019). LHTs were previously found to be positively charged carriers of the amino acids arginine, lysine, and histidine. Although the precise mechanism is still unknown, LHTs appear to be the primary transport vehicle in the ethylene pathway. The identification of ACC in the xylem suggests that it contributes to long-distance signaling and the systemic ethylene response (Yang et al., 2020). The interaction between ethylene and gibberellins forms a precisely regulated system that governs essential developmental processes, including root elongation and apical hook formation. Ethylene influences not only the biosynthesis and signaling pathways of gibberellins but also facilitates long-distance signaling via the transport of its precursor, ACC (1-aminocyclopropane-1-carboxylic acid). The discovery of amino acid transporters such as LHT1 and LHT2 as ACC carriers highlights the intricate nature of ethylene-related signaling pathways. Together, these mechanisms underscore the importance of ethylene–gibberellin crosstalk and ACC transport in balancing plant growth with environmental stress responses.

### 2.5 Abscisic acid

First identified as a growth inhibitor that builds up during fruit drop in the early 1960s, ABA has been linked to a variety of physiological functions, such as stomatal movement, seed germination, and reactions to environmental stressors. It is well known that ABA, in particular, acts as a hormonal stress signal throughout the plant, increasing resistance in distant organs. In response to variations in leaf hydration, guard cells are first and foremost autonomous for ABA synthesis. In addition, vascular parenchyma cells exhibit a significant increase in ABA biosynthesis in response to drought. Finally, one of the main causes of the accumulation of ABA content in roots after extended water stress is the basipetal transport of ABA from aerial organs. An optogenetics reporter specific to ABA that tracks cellular changes in dynamic ABA concentration in response to environmental stressors has validated these findings (Cotelle and Leonhardt, 2019).

The regulation of biosynthesis is impacted negatively by both GAs and ABAs, which must be balanced for a number of biological functions. However, dormancy and seed germination are particularly crucial also. Seed dormancy is avoided by ABA-INSENSITIVE4 (ABI4), which promotes ABA synthesis and GA2ox7 expression. Furthermore, during seed germination, ABA inhibits GA20ox2 and decreases GA levels via CHOTTO1 (CHO1) (Yano et al., 2009). The ABA/GA balance also takes into account a variety of external stimuli, such as temperature, light, and water stress, in order to maximize seed germination. The identification of multiple ABA transporters at the molecular level was done by Daszkowska-Golec (2022). It is significant that most of these transporters’ expression is found in close proximity to genes involved in ABA biosynthesis around vascular tissues, indicating that they may play a part in long-distance ABA transport, still, their individual roles in ABA transport at the acropetally and basipetal levels remain poorly understood (Kanno et al., 2012).

Transport mechanism: The production of ABA in the root and its subsequent transfer to the shoot has long been thought to mediate stomatal closure. It is now known, however, that the shoot, and more especially the vasculature, produces ABA (Jiang et al., 2025). Studies demonstrate that ABA activity at the stomatal aperture is restored by phloem-specific ABA synthesis. This implies that ABA might regulate various reactions (Merilo et al., 2015). The identification of a number of ABA transporters contributes to the understanding of the molecular mechanisms underlying ABA transduction. In *A. thaliana*, transporters belonging to the ABC family, including ABCG25 and ABCG40, are classified as ABA transporters. Guard cells import ABA through ABCG40, whereas the vasculature exports it through ABCG25. In order to protect cells, this implies that ABA is actively transported from the vasculature. ABA is transferred by ABCG25 and ABCG31 from the endosperm to the embryo, followed by ABCG30 and ABCG40 (Anfang and Shani, 2021) (**Figure 9**).

ABA transporters in *Arabidopsis* have also been identified as AIT1–4 and DTX50 (from the MATE family). Additional ABA transporters in other plants are also being described. To encourage stomatal closure, for example, it’s been demonstrated that the DELLA gibberellin repressor response is downstream of SlAIT1.1, an ABA importer in tomatoes (*Solanum lycopersicum* L.) (Shohat et al., 2020). The plasma membrane ABA transporters ABCG17 and ABCG18 redundantly mediate ABA import, mainly in leaf mesophyll cells. Their activity creates conjugated, inactive ABA sinks in mesophyll cells. This limits lateral root formation (long-distance ABA movement) and stomatal closure (short-distance ABA movement). Under normal conditions, ABCG17 and ABCG18 are essential for ABA homeostasis. However, during abiotic stress, their expression is suppressed. This releases free ABA, allowing a rapid stress response (Wong et al., 2023).

The ABA transporter ABCG25 is primarily expressed in the vascular veins of leaves and is located in the plasma membrane (**Figure 11**). ABA influences both seed dormancy and germination. In Arabidopsis, ABCG25 and ABCG31 transport ABA from the endosperm into the embryo. ABCG30 and ABCG40 then cooperate to import ABA into the embryo to induce seed dormancy (Kang et al., 2015). ABA transporters have also been identified in other species, including MtABCG20 in Medicago truncatula and LR34 in wheat. The ABA exporter MtABCG20 regulates nodule and lateral root growth (Pawela et al., 2019). ABA glucose ester (ABA-GE), the main conjugated form of ABA in xylem sap, may serve as the long-distance transport form (Hewage et al., 2020). Tonoplast-localized ABCC1 and ABCC2 are proposed to mediate ABA-GE uptake into vacuoles for inactive storage (Xu et al., 2012). Using this and other sensitive sensors can provide more insights into ABA movement and accumulation at both subcellular and whole-plant levels under stress and normal conditions (Zhou et al., 2024).Abscisic acid (ABA) serves as a key hormonal integrator of developmental cues and stress responses, regulated through tightly controlled biosynthesis and transport mechanisms. Its two-way movement between tissues is facilitated by a range of transporter proteins, including members of the ABC and MATE families such as ABCG25, ABCG40, AIT1s, and DTX50, allowing effective communication between roots and shoots under varying conditions. The regulation of ABA transport and conjugation plays a critical role in maintaining hormonal balance and enabling plants to swiftly respond to environmental changes. Recent developments in ABA-specific biosensors now provide valuable tools for mapping its distribution with high spatial and temporal resolution.

### 2.6 Gibberellins

Gibbrellins are diterpenoid chemicals that play an important role in plant growth, development, and flowering. GAs are mostly created near the site of action, however multiple investigations have shown that endogenously generated GAs may travel long distances. However, conflicting results have been reported for the mobile variant (Sirlin-Josserand et al., 2024). Inoculation tests in peas suggest that biologically inactive GA precursors may exist in mobile forms. Nonetheless, studies on maize show that bioactive GAs is transferred. The GA precursor GA12 is the predominant mobile form, as evidenced by micro-grafting tests between *Arabidopsis* hypocotyls from several GA-deficient mutants. The plant vascular system is also circulated by GA12, as demonstrated by quantitative analysis of endogenous GAs in xylem and phloem exudates (Regnault et al., 2015). In other words, GAs cannot pass through vascular tissues unless they reach particular tissues that need GA to grow. Genetic and biochemical studies have identified several GA transporters that are part of the NPF family (Tal et al., 2016).

In root elongating endodermal cells, the accumulation of bioactive GAs is facilitated by a plasma membrane entry transporter encoded by NPF3.1. This suggests that the root’s primary GA-responsive tissue is the endodermis. Fluorescently labelled bioactive GA4 is distributed differently in the root of NPF3.1 mutants due to the absence of NFP3.1 function. Nevertheless, it has no effect on root growth, indicating that GA transporters with redundant functions could make up for the lack of NPF3.1 (Tal et al., 2016). In elongating stamen filaments, bioactive GA3 has been demonstrated to be imported by a second GA transporter, NPF2.10/GTR1 (**Figures 9** and **11**). The application of exogenous GAs fully restores the phenotype to wild type, indicating that filament elongation and anther opening are significantly compromised in GTR1 mutants. Additionally, GTR1 can deliver the phytohormone jasmonoyl-isoleucine (JAIle), which is necessary for the development of stamens, into oocyte (Saito et al., 2015). Uncertainty surrounds GTR1’s role in the GA/JA interaction. It has been demonstrated that NPF4.1/AIT3 imports bioactive GAs into the heterologous system; however, its function has not been identified (Kanno et al., 2012). GAs are thought to offer a crucial organ-to-organ communication system that promotes adaptive growth throughout plant development, though the role of GA transport in regulating growth responses is not fully understood. This theory is supported by the fact that GAs is transported from the photosynthetic source to the recipient organs, where they aid in floral transition, internode elongation, and secondary stem growth (Dayan et al., 2012; Dietrich et al., 2025).

Transport mechanism: All vascular plants contain GA in a variety of forms, but only a few of these, including GA1, GA3, GA4, and GA7, are bioactive in plants. Other forms of GA are not bioactive; they are found in plants as catabolites or precursors. The genes encoding active GAs are expressed in different plant cells, tissues, and developmental stages, and their biosynthesis processes are complex (Castro-Camba et al., 2022). Evidence supports the transport of GA over short and long distances. According to Castro-Camba et al. (2022), the GA precursor GA12 is a mobile form that can travel from root to shoot. Additional markers for root-transported GA in *Arabidopsis* include tissue-specific expression of GA biosynthesis genes and differential fluorescence signaling of the GA-biosensor GPS1 between elongation and meristematic regions (Barker et al., 2021). The earliest known GA transporters are NPF proteins. NPF3 encourages GA efflux in the *Arabidopsis* root’s elongating endodermal cells. ABA stimulates its expression, while GA and nitrogen suppress it (Tal et al., 2016). The GA transporter function appears to be a function of the NPF transporter GTR1 (also called NPF2.10) in *Arabidopsis*. Further research is required to identify missing GA exporters and potentially other importers that control GA movement during germination and flowering.

Furthermore, the DELLA protein RGA is the basis for a ratiometric GA signaling biosensor that detects changes in GA transport in the plant and reports cellular changes in GA content in the shoot apical meristem. It has been shown that the transcription factors TEM1 and TEM2 negatively regulate the expression of the GA transporter specific to NPF (GTR1, NPF3, and NPF2.3), which in turn controls the exclusive accumulation in mesophyll cells that occurs as a result of the exogenous application of fluorescently labelled GA3. As a result, mesophyll cells exhibit variable GA accumulation and distribution, which controls trichome initiation in the epidermis and lends credence to the idea that GA migrates between plant tissues (Matías-Hernández et al., 2016). Gibberellins (GAs) play a central role in controlling plant growth, developmental transitions, and organ differentiation through both localized signaling and long-distance movement. GA_12_ is recognized as the main mobile precursor facilitating inter-tissue transport. Members of the NPF transporter family, including NPF3.1, NPF2.10/GTR1, and AIT3, are crucial for mediating GA uptake and distribution throughout the plant. The regulation of GA transport is highly dynamic and influenced by external and internal cues such as abscisic acid (ABA) and nitrogen availability, enabling fine-tuned hormonal balance for developmental processes like stem elongation, root growth, and flowering. Advances in GA-specific biosensors and molecular tools are shedding new light on the spatial and temporal regulation of GA signaling and movement.

### 2.7 Jasmonates

Lipid-derived molecules called JAs (oxylipins) regulate a number of developmental processes, including root elongation and fertility as well as plant adaptation to environmental stressors like pathogens, insect herbivory, and wounding (Li C. et al., 2022). The first JA was identified in 1957 and was cis-jasmone, a fragrant component of *Jasminum grandiflorum* essential oil. Most famously, JAs play a role in vegetative and reproductive processes like root elongation and fertility, as well as plant adaptation to environmental stimuli, including biotic and abiotic stresses (Wasternack and Strnad, 2018). Both local and systemic defense responses are triggered by the production of JAs in response to tissue damage. As a result, JAs are advised to relocate and work outside of their synthesis location.

While studies in *Arabidopsis* indicate the presence of a transmitted wound signal that initiates systemic synthesis of JAs, labelled JA feeding and inoculation experiments in *Solanaceae* seem to support long-distance transport of JAs (Jacobs et al., 2016). GLUTAMATE RECEPTOR-LIKE (GLR) genes allow damaged leaves to show their damage status by sending electrical signals to undamaged leaves, which in turn stimulates distal JA production and signaling (Mousavi et al., 2013). Conversely, damaged leaves generate JAs in xylem contact cells, which travel long distances, most likely through the plant vascular system, from cell to cell before arriving at undamaged tissues. GLR genes enable damaged leaves to communicate their damage status to undamaged leaves through electrical signals, which in turn triggers the production and signaling of distal JA (Larrieu et al., 2015; Gasperini et al., 2015). Many cells have high levels of JA and JA-Ile (the biologically active form) following injury, which suggests that they could be used for mobile signaling. Nevertheless, the type of JA that is mobile is still unknown.

Transport mechanism: Chloroplasts are the site of the first half of JA biosynthesis, which yields the intermediate cis-12-oxophytodienoic acid (OPDA). A START protein called JASSY, which is found in the chloroplast outer envelope membrane, has been demonstrated to export OPDA from chloroplasts (Guan et al., 2019). According to recent reports based on inoculation experiments, OPDA and its derivatives must pass through the phloem, but not the bioactive JA-Ile conjugate, in order to initiate JA signaling in the root. The peroxisomal membrane contains CTS, also referred to as ABCD1, which makes sure that OPDA is delivered to the peroxisomes, where further JA biosynthesis occurs (Schulze et al., 2019).

JA-Ile, which is thought to be the bioactive form of JA, is detected by the nucleus. Consequently, it is intriguing that ABCG16 (also called JAT1) mediates the nuclear efflux of JA-Ile as well as the cellular efflux of free JA molecules and localizes to both the nuclear envelope and the plasma membrane (**Figure 11**). As far as we know, this was the first instance of a nuclear-localized hormone transporter in plants. JAT3 and JAT4, which are *Arabidopsis* ABCG6 and ABCG20, respectively, actively control the flow of JA from leaf to leaf that is generated in response to wounding. Thus, two JA and low-affinity JA-Ile importers that are expressed in the phloem and present in the plasma membrane work together to facilitate long-distance JA translocation. Since NPFs can help transport ABA, glucosinolates, and GA, their transport of JA is non-specific (Lacombe and Achard, 2016). Since these plant transporters are members of a large protein family, genetic redundancy most likely severely restricts their physiological characterization. jasmonates (JAs) function as crucial lipid-derived hormones that regulate plant growth, defense mechanisms, and wound responses through both localized activity and systemic transport. Precursors like OPDA, originating in the chloroplast, and its derivatives are transported via the phloem, facilitating long-distance signaling. Transport proteins such as JASSY, CTS (also known as ABCD1), and ABCG family members including JAT1, JAT3, and JAT4 enable the movement of JAs across cellular membranes and tissues. The presence of these transporters at the nuclear envelope and plasma membrane supports tight control of JA signaling pathways. Although significant progress has been made, the exact identity of the primary mobile JA form and the full scope of its transport mechanisms remain unresolved.

### 2.8 Strigolactones

The molecules known as SLs were initially discovered to be produced by parasitic plants. However, they were later found to be endogenous phytohormones. It appears that cross-talk between SLs and GAs controls sister bud protrusion and shoot elongation (Nelson, 2021). The interaction between the SLR1 and DWARF 14 (D14) proteins depends on SLs. Conversely, genes involved in SL biosynthesis are suppressed by GA signaling. GAs stimulates bud growth in *Jatropha curcas*, while SLs inhibit it (Yang et al., 2019). SLs also regulate the ABA/GA ratio by reducing ABA synthesis. In rice, SLs control shoot elongation, affecting GA signaling and metabolism (Yang et al., 2019). Strigolactones, derived from carotenoids, regulate multiple developmental processes and modify plant architecture according to nutrient availability. SLs released from roots into the soil by arbuscular mycorrhizal fungi promote root colonization, helping plants absorb more nutrients (Chen et al., 2022). SLs are also transported from root to shoot to suppress lateral bud growth (Huizhi et al., 2022).

According to grafting studies, SLs are made in both roots and shoots, and the xylem transports SL precursors to the shoot to regulate shoot architecture (Seto and Yamaguchi, 2014). Wild-type rootstocks can give grafted SL-deficient petioles their wild-type developmental habit, while wild-type shoots display a wild-type phenotype when grafted on SL-deficient rootstocks, indicating that shoots are autonomous for SLs (Tejada-Alvarado et al., 2022). It has been established that the G-type ABC transporter known as petunia’s pleiotropic drug resistance 1 (PDR1) is crucial for the release of SLs from the vascular and nodal tissues surrounding axillary buds as well as from cells that produce roots. In root hypodermal cells, PDR1 is asymmetrically localized and accumulates in shoot axils (Jianchao et al., 2023). According to Zhang Y. et al. (2023), it thus facilitates mycorrhizal fungal entry as well as the acropetally transport of SLs. The lateral branching phenotype of PDR1 mutants is consistent with its role as a cellular SL exporter. Because of their deficiencies in SL exudation in soil, these PDR1 mutants have fewer symbiotic relationships.

**Transport mechanism:** It has been discovered that SLs control the regeneration and formation of vascular tissue by blocking auxin PIN-dependent feedback transport. The SL transporter ABCG class protein PDR1 is an exporter that is expressed in the root cortex and shoots axes and is located in the plasma membrane (Alvi et al., 2022) (**Figure 10**). Hypodermal, root axillary, and root tip cells’ short-distance transport is regulated by PDR1, according to experiments. That means it’s also involved in SL’s long-distance transportation. But PDR1 might not be the only factor in SL long-distance transport. More transporters and SL precursors may be on the hunt (Shiratake et al., 2019; Qianqian et al., 2022). Whether SL compounds move within plants and whether other plant species have PDR-mediated transport are still unknown. The original identification of SLs as parasitic weed germination stimulants raised doubts about why plants release substances that could be harmful to their survival. Graft studies on *A. thaliana*, primarily by the Domagalska and Leyser group (Hartung et al., 2002), demonstrate that both shoots and roots synthesize SLs through shared players. The mutant phenotype can be improved and SL supplied to the pen by grafting wild-type roots onto a mutant shoot lacking SL biosynthesis. These investigations have led to the conclusion that SLs need to adhere to two transport pathways. They must be transported in shoot directions to control shoot architecture, and they must be expelled from the roots to start hyphal branching. The SL transporter might be a member of this ABC family, given that ABCG transporters have been demonstrated to transport terpenoids and that this class of proteins is thought to be involved in biotic and abiotic stress responses (Hedden, 2020; Yangwenke et al., 2022).

GR24, a synthetic SL, and phosphate starvation, a condition known to induce SL excretion, were found to up-regulate root-expressed ABCGs in *Petunia hybrida*, which they used as a model system because many ABCs are known to be up-regulated by their substrates (**Figure 9**). Petunia PDR1, one of the candidate genes, satisfies these criteria and is undergoing additional analysis. PDR1 is expressed at the tissue level in stem nodes close to the axillary buds and the root vasculature, as well as in hypodermal transition cells (HPCs) and root tips. The HPCs have received particular attention because mycorrhizal fungi are known to enter through them (Hedden and Sponsel, 2015; Yinfeng et al., 2012). To find out if petunia PDR1 is actually an SL exporter, the authors expressed it in the distantly related species *A. thaliana*, a plant with low endogenous SL-exuding activity and no SL functional homologue so far found (Shoji et al., 2023). In addition to being more resistant to synthetic SL GR24 supplements, *Arabidopsis* plants overexpressing PDR1 also excreted significantly more GR24 than the corresponding wild type when preloaded with the SL via radioactive labeling. The fact that petunia PDR1 is an SL transporter is confirmed by these findings. Although PDR1 petunia mutants exhibit a phenotype that is clearly weaker than biosynthesis mutants, they exhibit stronger shoot lateral branching aboveground than the corresponding wild type (Dong et al., 2024). HPCs were found to express NtPDR6, the tobacco homologue that is most similar to petunia PDR1. Furthermore, plants that have NtPDR6 silenced have a bushy phenotype. The purpose of a study is to better understand the movement of SLs from the root tip, where they are synthesized, to the soil and shoots. The localization of a GFP-PDR1 fusion construct expressed by plants. Different root tissues have asymmetric and cell type-specific localization of PDR1 (Hwang et al., 2012). PDR1, which was co-expressed with the SL biosynthesis gene and found in the apical membrane of root hypodermal cells, decreased apical dominance1 (DAD1/CCD8) in root tips, possibly enabling SL transport to the shoot. Furthermore, in plants that over expressed PDR1, the auxin transporter PIN1’s protein levels were significantly down-regulated in the root tip stele, while PIN2 was either unaffected or even stimulated. These findings support the notion that SL and auxin transport are closely related, as indicated by earlier research that applied SLs exogenously to *Arabidopsis* roots and shoots (Dun et al., 2023). Overall, strigolactones (SLs) are essential plant hormones that control shoot branching, mediate root-to-shoot signaling, and facilitate symbiotic relationships with arbuscular mycorrhizal fungi. The ABCG transporter PDR1 is a key mediator of SL export from roots and nodal regions, supporting both localized action and systemic transport via the xylem. Its asymmetric distribution and upregulation under phosphate-deficient conditions underscore the role of SLs in plant adaptation to nutrient scarcity. Despite current insights, additional transporters and unidentified SL precursors are likely involved in the broader regulatory network governing SL movement and signaling throughout the plant.

### 2.9 Melatonin

The effects of the well-known hormone melatonin on plant development, such as how it affects biomass production, seed germination, and photosynthetic efficiency, have only lately come to light (Naz et al., 2024). Cotton seed germination is improved by exogenous melatonin treatment, which concurrently raises GA content and lowers ABA levels. Even in situations with high salinity, melatonin in cucumbers promotes seed germination by activating GA20ox and GA3ox (Castro-Camba et al., 2022). By regulating GA levels, melatonin increases plants’ ability to withstand stress. Melatonin pretreatment boosts tomato tolerance and GA20ox expression under heat stress (Ali et al., 2024) (**Figure 11**). However, when watermelon plants are exposed to cold stress, melatonin treatment creates a tolerance response and lowers GA levels. Melatonin functions as a multifunctional regulator of plant growth and stress adaptation by modulating gibberellin (GA) homeostasis. In rice, a deficiency in melatonin leads to reduced brassinosteroid (BR) levels, which in turn lowers GA content, contributing to increased tolerance to cadmium, cold, salt, and heat stress (Madebo et al., 2024). This indicates that melatonin-triggered stress responses are closely linked to the regulation of GA levels. Melatonin promotes germination, biomass accumulation, and stress resilience by upregulating GA biosynthetic genes such as GA20ox and GA3ox, while also coordinating with abscisic acid (ABA) and BR signaling pathways. Depending on the type of stress encountered, melatonin can either enhance or suppress GA levels to optimize physiological responses, emphasizing its role as a key modulator of hormonal crosstalk in plants.

### 2.10 Karrikins

Karrikins, smoke-derived compounds, generally promote seed germination in various species. In Arabidopsis thaliana, they enhance the expression of GA biosynthesis genes such as GA3ox1 and GA3ox2, suggesting a GA-dependent role in germination (Waters and Nelson, 2023). However, their effects are context-dependent. In soybean, karrikins reduce GA synthesis and increase ABA levels, delaying germination in dark conditions (Wang et al., 2018). Karrikins also play a protective role under abiotic stress by preventing germination in unfavorable environments (Wang et al., 2018; PMC7105677). Overall, karrikins modulate the ABA/GA balance, integrating environmental cues to control germination and early seedling development, highlighting their complex and species-specific regulatory functions. Karrikins are powerful regulators of seed germination that modulate the balance between abscisic acid (ABA) and gibberellins (GA) in response to environmental signals. They influence germination by either enhancing or repressing the expression of GA biosynthetic genes, depending on the species—for example, promoting GA production in Arabidopsis while inhibiting it in soybean. This selective regulation ensures that germination is restricted to favorable environmental conditions. In addition to their role in germination, karrikins also support early stress avoidance mechanisms, highlighting their context-dependent and species-specific function in plant developmental processes.

### 2.11 Gamma-aminobutyric acid

Recent research has revealed that plants contain significant levels of γ-aminobutyric acid (GABA), a non-protein amino acid that functions as a key signaling molecule in diverse physiological and developmental processes, including stress perception, carbon–nitrogen balance, and reproductive development. Although the interaction between GAs and GABA remains relatively underexplored, emerging evidence suggests a functional association between these two signaling molecules. For instance, exogenous application of GAs has been shown to modulate GABA accumulation in several plant species, with the response being dependent on the developmental stage and timing of application. In grapevines, GA treatment enhances GABA levels in developing tissues, while in rice, GA application increases GABA content in seeds, suggesting that GA may influence GABA metabolism through transcriptional or post-translational regulation of GABA biosynthetic enzymes (Sun et al., 2024). Similarly, under drought stress, *Auricularia fibrillifera* exhibits simultaneous upregulation of genes associated with GA biosynthesis and GABA metabolism, implying a potential synergistic interaction that contributes to stress mitigation and recovery (Wang et al., 2022). Additionally, studies in *Arabidopsis thaliana* and *Solanum lycopersicum* have indicated that GABA accumulation can influence GA signaling pathways, possibly through modulation of DELLA protein stability and hormone cross-regulation. Collectively, these findings suggest that GABA and GAs may act in concert to fine-tune growth and stress responses, representing an emerging layer of hormonal and metabolic crosstalk in plants. Recent studies identify GABA as a vital non-protein amino acid regulating plant growth, stress perception, and C-N balance. Evidence suggests functional interplay between GABA and GAs, as GA treatments enhance GABA accumulation and modulate its biosynthetic enzymes across species. Conversely, GABA appears to influence GA signaling, possibly via DELLA protein regulation. Together, GABA and GAs form an emerging crosstalk network fine-tuning plant growth and stress adaptation.

## 3 Physiological role of various phytohormones in plant membrane transport regulation

The meristem is one of the most important sites for auxin transport activity in relation to plant growth and development. It can be viewed as a reverse auxin fountain that flows from vascular tissue towards the root and is redirected through the meristem epidermis towards the shoot (Peer et al., 2011). It is believed that auxin released from cells going through programmed cell death moves radially inward and contributes to the periodic peaks that determine the location of lateral roots (Yalamanchili et al., 2024). The elements of the outward flow that control the development of lateral roots, however, are poorly understood. According to a recent study, the root’s outer tissues lack auxin transporters. By localizing to the plasma membrane, transporting auxin extracellularly, and acting redundantly, these five genetically linked ABCB transporters move IAA through the lateral root cap and epidermis to the maturation zone, enabling lateral root spacing (Chen et al., 2023).

To initiate guard cell reactions, ABA biosynthesis from phloem companion cells is sufficient. ABA may maintain positive leaf turgor in this way. Numerous lines of evidence indicate that loss of turgor, rather than exceeding a certain water potential or solute potential threshold, is the signal for ABA production (Kane and McAdam, 2023). This would raise plant turgor and aid in maintaining homeostasis in that parameter if ABA production in this manner resulted in stomatal closure. The loss of leaf turgor, however, is a rare physiological phenomenon that is most likely caused by presumed root spatial signaling (Bharath et al., 2021). Similar to long-distance CK transport from root to shoot, local CK transport is known to impact plant growth and development. The identification of the plasma membrane-localized H+-bound high-affinity purine permiator PUP14 as a CK importer in *Arabidopsis* highlights the importance of the biochemical transport process (**Figures 9** and **10**). Because plants cannot make it through the embryonic stage without this activity (Tessi and Maurino, 2024). CK regulates cell-to-cell transport and impacts CK homeostasis by managing CK long-distance transport and local allocation (Xiao et al., 2019). As a result, the PUP CK transporter family controls the growth and development of plants.

GA transport is concluded to be crucial for the formation of suberin in the root, a particular developmental context. Suberin formation is regulated in concert by one tonoplast importer, NPF2.14, and two new GA and ABA importers, NPF2.12 and NPF2.13. These findings fill in the gap in the mechanism underlying GA12’s long-distance transport. Vacuolar accumulation of GA and ABA begins in the phloem unloading zone surrounding the root elongation zone, where the two hormones are stored in vacuolar storage as the root matures and differentiates. According to these results, GA and ABA regulate plant development in a non-antagonistic manner (Li M. et al., 2022). The new mechanism also provides the first explanation of the developmental significance of long-distance GA12 shoot-root movement and the biological significance of bioactive GA4 and ABA accumulation in the endodermis to regulate endodermal suberization.

Grafting studies and analysis of exogenous radiolabeled JA and JA-Ile have demonstrated that JAs can serve as transmissible wound signaling from the local damage site to systemic leaves. However, the precise molecular form transported has not yet been identified (Li et al., 2020). Conditions that change how easily ethylene diffuses from its production site to its target sites can significantly impact the ethylene response, even though active transporters do not control this process. For instance, it has been demonstrated that, in comparison to loose soils, compacted soils permit substantially less gas diffusion because they have fewer air-filled pores. This causes ethylene to build up in root tissues and sets off hormonal reactions that limit the growth of *Arabidopsis* (Pandey et al., 2021). Consequently, roots can use the volatile ethylene hormone as an early warning system to steer clear of compacted soils. The ethylene response in compacted soils requires ABA and auxin biosynthesis as well as AUX1-mediated auxin transport, and studies on rice have shown that the mechanism is conserved in monocotyls (Pandey et al., 2021). The rate at which the hormone diffuses out of the plant in response to the external concentration tells the responding tissues about the current conditions. To prove this function, a sensing system that can react to external concentration and proof of environmentally significant variations in diffusion rates are needed. This process has been suggested to function during the emergence of dicot seedlings that are germinating, when the gaseous hormone ethylene is thought to regulate growth patterns (Moulton et al., 2020). The plant continuously produces the gas, which diffuses into soil air spaces. The concentration of the gas determines whether the plant is constrained by the soil (high concentration) or diffuses into free air (low concentration). The amount of ethylene has an impact on the thickness and growth rate of epicotyls as well as the rate at which the plumular hook does not curl. This sensing system is linked to another system that uses phytochrome to respond to light quality as an indicator of emergence. Additionally, the ethylene and IAA response systems may be able to communicate (Bertoni, 2020). This category also includes seed coat inhibitors, whose diffusion away from the seed increases during wet conditions; this could reveal the soil’s water status and suggest a good time for germination (Upretee et al., 2024).

Only when growth conditions are unfavorable (i.e., light, nutrition, water, and oxygen supply imbalances) do plant hormones become significant regulators. These reactions, according to researchers, are caused by “metabolic sensitization,” or acquired sensitivity to the hormone of interest as a result of the altered circumstances. This role requires a change in sensitivity following an imposed stress, possibly in combination with changes in hormone concentration. These variables cause sensitization or combined control, which can be used to measure the growth or developmental response that stresses causes. The response ought to reduce the stress to some extent. One example in this category is how ethylene affects how plants react to flooding and waterlogging. By restoring leaf air contact, this response raises the likelihood of survival and includes both ethylene concentration changes and the difference in ethylene sensitivity between young and old leaves (Li et al., 2021). Plants can communicate with one another through pheromones and allele-chemicals. Since the compound is likely to have a strong local effect that diminishes significantly with distance, evidence that it can elicit a response at concentrations commonly found in the environment is required to demonstrate such an effect. Particularly for allele-chemicals, removing additional potential controlling effects can pose logical and experimental challenges (Friesen et al., 2024). The behavior pattern must have a measurable positive impact on the signaling individual or gene pool. Allelopathy frequently involves preventing another plant’s growth, metabolism, pollen, pores, or seeds from germinating (Rizvi et al., 1992), and it goes without saying that this will lessen competition. A large number of phenolics are among the many allele-chemical compounds. Higher plants may experience a warning component from the pheromone effect, such as insect attack, and may respond by reducing herbivory. According to researchers, methyl salicylate in *Nicotiana* and methyl jasmonate in *Artemisia* has pheromone functions. Through the regulation of physiological processes, hormones enable plants to accelerate or decelerate biological time, or “biotime”. The inverse relationship between development and temperature is comparable to this idea. In this case, the scale of developmental biotime varies with the current temperature. Researchers proposed that biospan might be similarly impacted by hormone concentration. It will be necessary for researchers to explicitly outline the role that is assumed, the additional chemical control criteria that will function in this system, and how the specific piece of evidence that has been reported fits into the applied framework (Weyers and Paterson, 2001).

## 4 Conclusion

Membrane transporters in plants play a vital role in maintaining cellular equilibrium by facilitating the movement of ions, nutrients, and phytohormones across cellular membranes. Recent research highlights a reciprocal relationship between transporters and phytohormones: while hormones influence the expression, localization, and activity of transporters, these transporters, in turn, regulate hormone distribution and signaling intensity. This mutual regulation is orchestrated through multiple mechanisms, including transcriptional changes, protein modifications such as phosphorylation and ubiquitination, and vesicle-mediated transport. Such coordination allows plants to modulate their growth and stress responses with precision. Although significant progress has been made in this field, our understanding remains incomplete due to the narrow focus of many studies on individual transporters or specific hormones. To achieve a more comprehensive view, future research must integrate spatial and temporal dynamics of hormone transport. This will require the use of live-cell imaging, biosensors for hormone tracking, and multi-omics technologies to observe how transporter systems respond to multiple or sequential environmental stresses. Furthermore, innovations like CRISPR/Cas-mediated genome editing and synthetic biology hold promise for designing optimized transporter pathways that could improve plant nutrient efficiency and stress tolerance. Exploring the role of transporters in facilitating hormonal exchange between roots and shoots, as well as in interactions with the rhizosphere, may also offer novel avenues for crop enhancement. In summary, hormone-transporter systems operate as interconnected, flexible networks that align plant growth with environmental signals. Deepening our understanding of these interactions is crucial for developing resilient crop varieties capable of withstanding the multifaceted challenges posed by climate change.
